# Development of a CRISPR/Cas9 genome editing toolbox for *Corynebacterium glutamicum*

**DOI:** 10.1186/s12934-017-0815-5

**Published:** 2017-11-16

**Authors:** Jiao Liu, Yu Wang, Yujiao Lu, Ping Zheng, Jibin Sun, Yanhe Ma

**Affiliations:** 1 Key Laboratory of Systems Microbial Biotechnology, Chinese Academy of Sciences, Tianjin, 300308 People’s Republic of China; 2 Tianjin Institute of Industrial Biotechnology, Chinese Academy of Sciences, Tianjin, 300308 People’s Republic of China

**Keywords:** CRISPR/Cas9, Genome editing, *Corynebacterium glutamicum*, Plasmid-borne templates, Gene deletion/insertion, Single-nucleotide editing, Double-locus editing

## Abstract

**Background:**

*Corynebacterium glutamicum* is an important industrial workhorse and advanced genetic engineering tools are urgently demanded. Recently, the clustered regularly interspaced short palindromic repeats (CRISPR) and their CRISPR-associated proteins (Cas) have revolutionized the field of genome engineering. The CRISPR/Cas9 system that utilizes NGG as protospacer adjacent motif (PAM) and has good targeting specificity can be developed into a powerful tool for efficient and precise genome editing of *C. glutamicum*.

**Results:**

Herein, we developed a versatile CRISPR/Cas9 genome editing toolbox for *C. glutamicum*. Cas9 and gRNA expression cassettes were reconstituted to combat Cas9 toxicity and facilitate effective termination of gRNA transcription. Co-transformation of Cas9 and gRNA expression plasmids was exploited to overcome high-frequency mutation of *cas9*, allowing not only highly efficient gene deletion and insertion with plasmid-borne editing templates (efficiencies up to 60.0 and 62.5%, respectively) but also simple and time-saving operation. Furthermore, CRISPR/Cas9-mediated ssDNA recombineering was developed to precisely introduce small modifications and single-nucleotide changes into the genome of *C. glutamicum* with efficiencies over 80.0%. Notably, double-locus editing was also achieved in *C. glutamicum*. This toolbox works well in several *C. glutamicum* strains including the widely-used strains ATCC 13032 and ATCC 13869.

**Conclusions:**

In this study, we developed a CRISPR/Cas9 toolbox that could facilitate markerless gene deletion, gene insertion, precise base editing, and double-locus editing in *C. glutamicum*. The CRISPR/Cas9 toolbox holds promise for accelerating the engineering of *C. glutamicum* and advancing its application in the production of biochemicals and biofuels.

**Electronic supplementary material:**

The online version of this article (10.1186/s12934-017-0815-5) contains supplementary material, which is available to authorized users.

## Background

The Gram-positive soil bacterium *Corynebacterium glutamicum* was discovered about 60 years ago, and was originally well-known as an excellent producer of glutamate [1]. With the development of biotechnology, *C. glutamicum* has been successfully engineered to serve as a versatile workhorse for industrial bioproduction. Nowadays, this bacterium is used to produce more than 4 million tons of diverse amino acids per year and a wide range of other natural and non-natural products, which are used as feed additives, nutritional supplements, pharmaceutical intermediates, biofuels, and polymer building blocks [2]. It is estimated that products generated via *C. glutamicum* fermentation will reach a market size of US$20.4 billion by 2020 [3].

At the early stage of engineering of *C. glutamicum*, random mutagenesis combined with positive selection by phenotypic resistance to amino acid analogs was the most commonly used strategy [4]. Genetic manipulations in *C. glutamicum* were initiated in 1984 and became a key enabling strategy for strain improvement [5]. A routinely used method for gene disruption and insertion in *C. glutamicum* is based on integration of a suicide vector into its chromosome, followed by a second recombination event to remove the plasmid backbone and a counter-selection step using a conditionally lethal marker. Nevertheless, due to the frequent spontaneous inactivation of the counter-selectable marker *sacB*, up to 45% of colonies obtained in the screening process were false-positive, making this multi-step procedure time-consuming and inefficient [6].

Recently, clustered regularly interspaced short palindromic repeats (CRISPR) and their CRISPR-associated proteins (Cas) have been explored as a leading-edge tool for genome editing in eukaryotic host cells, plants, and animal models of human disease [7–9]. Although CRISPR/Cas systems (especially CRISPR/Cas9 systems) are derived from bacteria or archaea, their applications have not been extensively employed in bacteria [10]. To date, successes of bacterial genome editing using CRISPR/Cas9 systems have been reported in limited bacteria including *Escherichia coli* [11–19], *Streptococcus* species [11, 20], *Lactobacillus reuteri* [21], *Streptomyces* species [22–24], *Tatumella citrea* [12], *Clostridium* species [25–29], *Bacillus subtilis* [30–32], *Myceliophthora* species [33], and *Synechococcus elongates* [34].

To engineer *C. glutamicum* more efficiently and unleash its potential in industrial biotechnology, facile yet robust genome editing tools are urgently demanded. The CRISPR/Cas9 system that utilized NGG as protospacer adjacent motif (PAM) and has good targeting specificity is expected to enable genome-wide scale and precise editing of GC-rich *C. glutamicum* [10]. To our knowledge, there were many tries in the community to adapt CRISPR/Cas9 into *C. glutamicum* for comprehensive genome editing. In the time this manuscript was being prepared, Cho and coworkers reported CRISPR/Cas9-mediated genome editing of *C. glutamicum* using *cas9* gene codon-optimized for its use in actinomycetes and *recT* gene encoding *E. coli* prophage recombinase [35]. Without the use of *recT*, no positive transformants could be obtained using double-stranded DNA (in both linear and replicative plasmid form) or single-strand DNA (ssDNA) as editing templates. Further introduction of *recT* facilitated deletion of 400-bp chromosomal fragments using ssDNA as editing templates. However, Cho and coworkers didn’t report whether the optimized CRISPR/Cas9 system including RecT could delete or insert genes into the chromosome of *C. glutamicum* with plasmid-borne templates [35]. Meanwhile, Jiang and coworkers independently developed a CRISPR/Cpf1-mediated genome editing tool for *C. glutamicum* [36]. The CRISPR-Cpf1 system combined with ssDNA recombineering can efficiently introduce small changes into the *C. glutamicum* genome. Large gene deletions and insertions are also realizable using this system [36]. However, because *Francisella novicida* Cpf1 utilizes a T-rich PAM [37], its editing targets in GC-rich *C. glutamicum* genome are supposed to be fewer than *Streptococcus pyogenes* Cas9. Therefore, development of a powerful CRISPR/Cas9-mediated genome editing toolbox that can delete, insert and modify genes in *C. glutamicum* flexibly and multiply is still urgently demanded.

In this study, we successfully developed a CRISPR/Cas9 toolbox for efficient and comprehensive engineering of several *C. glutamicum* strains. By using the tailor-made CRISPR/Cas9 system, gene deletion and insertion with plasmid-borne editing templates were achieved with efficiencies of 30.8–60.0% and 16.7–62.5%, respectively. CRISPR/Cas9-mediated ssDNA recombineering was developed to introduce small modifications and single-nucleotide changes into the genome with efficiencies over 80.0%. Double-locus editing was also realized in *C. glutamicum* with an efficiency of 40.0%. The toolbox developed here is simple and versatile, which will provide solutions to overcome major limitations of existing genome editing technologies of *C. glutamicum* and advance engineering and application of this industrial workhorse.

## Results

### Optimization of Cas9 and gRNA expression for lethality-based selection

It has been repeatedly reported that the double-strand breakage (DSB) induced by Cas9 is lethal to bacterial cells because many microorganisms lack the endogenous nonhomologous end joining (NHEJ) mechanism, or the NHEJ is not efficient enough to repair the DSB. As a result, CRISPR/Cas9 was usually used as a lethality-based selection tool in bacterial cells [25, 26]. To achieve CRISPR/Cas9-mediated genome editing, the lethality of Cas9-induced DSB was first evaluated.

**Fig. 1:**
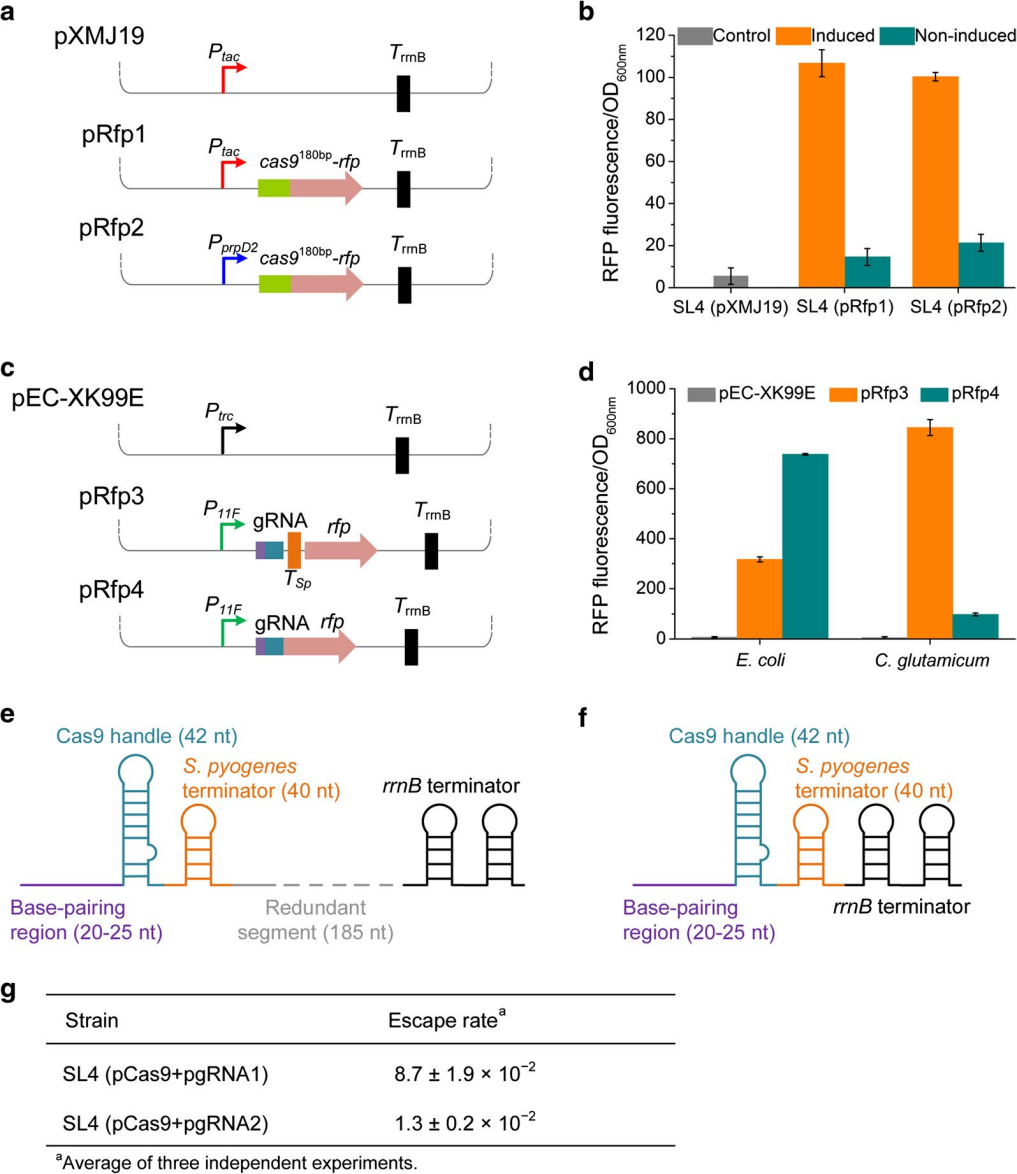
Optimization of CRISPR/Cas9 system in *C. glutamicum*. **a** Schematic representation of the plasmids used for optimizing the promoter of Cas9 expression. The *cas9* ^180bp^-*rfp* fusion gene that consisted of the first 180 bp of *cas9* gene and the full-length *rfp* gene was inserted into pXMJ19 under the control of IPTG-inducible promoter *P*_*tac*_ (pRfp1) or propionate-inducible promoter *P*_*prpD2*_ (pRfp2). The empty plasmid pXMJ19 was used as a negative control. **b** Optimization of promoters for Cas9 expression. pRfp1, pRfp2, and pXMJ19 were transformed into *C. glutamicum* SL4 separately. The resultant transformants were cultivated in SGY medium with or without 1 mM IPTG (for pRfp1) or 1 g/L sodium propionate (for pRfp2). Cells of the stationary growth phase were used to detect their fluorescence outputs using a microplate reader (λ excitation = 560 nm, λ emission = 607 nm). **c** Schematic representation of the plasmids used for verifying the function of the terminator derived from *S. pyogenes* (*T*_*Sp*_). A *rfp* gene was inserted downstream the gRNA (pRfp3) and the modified gRNA with *T*_*Sp*_ deleted (pRfp4). The empty plasmid pEC-XK99E was used as a negative control. **d** Function verification of *T*_*Sp*_ in *E. coli* and *C. glutamicum* by detecting fluorescence outputs of strains harboring pRfp3, pRfp4, or pEC-XK99E. **e** The gRNA structure derived from plasmid pgRNA1. **f** The optimized gRNA structure derived from plasmid pgRNA2. **g** Escape rate of CRISPR/Cas9-based counter-selection using different gRNA expression plasmid. pgRNA1 and pgRNA2 were transformed into *C. glutamicum* SL4 (pCas9) separately. Correct transformants were cultivated, diluted and spread on SGY plates containing Km and Cm, with or without IPTG (1 mM). The escape rate of counter-selection was calculated by colony counting

Constitutive expression of dCas9 from *S. pyogenes* in *C. glutamicum* has been proven unattainable [3]. In a previous study, isopropyl-β-d-thiogalactopyranoside (IPTG)-inducible promoter *P*_*tac*_ and propionate-inducible promoter *P*_*prpD2*_ were used for dCas9 expression, and *P*_*tac*_ was used for gRNA expression, facilitating CRISPR interference (CRISPRi) in *C. glutamicum* [3]. Inspired by this study, we used the same biological parts to prepare Cas9 and gRNA expression cassettes. Strictly controlled expression of Cas9 was considered beneficial for screening desirable mutants when using CRISPR/Cas9 for gene deletion [26]. To optimize the promoter for Cas9 expression, *P*_*tac*_ and *P*_*prpD2*_ were employed to drive the expression of a Cas9-red fluorescent protein (RFP) fusion protein that consisted of the first 180 bp of *cas9* gene and the full-length *rfp* gene. The artificial *P*_*tac*_/*P*_*prpD2*_-*cas9* ^180bp^-*rfp* cassettes were inserted into pXMJ19, generating plasmids pRfp1 and pRfp2, respectively (Fig. 1a). Fluorescence outputs of the engineering cells harboring pRfp1 and pRfp2 were detected in the presence or absence of inducers. The results demonstrated that *P*_*tac*_ was controlled more strictly by IPTG than *P*_*prpD2*_ by sodium propionate (Fig. 1b). Plasmid pCas9 was then constructed by inserting *P*_*tac*_-*cas9* cassette into pXMJ19 and transformed into *C. glutamicum* SL4, a derivative of strain ATCC 13869 with high electrotransformation efficiency, generating strain SL4 (pCas9). The gRNA targeting on the *C. glutamicum* lactate dehydrogenase gene *ldhA* was designed and inserted into pEC-XK99E under the control of *P*_*tac*_, resulting in pgRNA1. Transformation of pgRNA1 into strain SL4 (pCas9) was performed and cells were spread on soya peptone-glucose-yeast extract (SGY) plates containing kanamycin (Km) and chloramphenicol (Cm) with or without 1 mM IPTG. All the colonies growing on the plates were counted to calculate the escape rate. A high escape rate (8.7 ± 1.9 × 10^−2^) was obtained, which was unacceptable for lethality-based selection.

The unoptimized gRNA expression cassette may be one reason for the high escape rate of CRISPR/Cas9-based counter-selection. Considering that *cas9* and gRNA were both under control of *P*_*tac*_, *P*_*11F*_ which is a derivative of the strong constitutive promoter *P*_*cspB*_ of *C. glutamicum* [38] was recruited for gRNA expression to avoid possible interference between the two *P*_*tac*_ promoters. The secondary structure of gRNA is also crucial for forming Cas9-gRNA complex. The gRNA used here consists of three domains: a 20–25 nt complementary region for specific DNA binding, a 42 nt hairpin for Cas9 binding (Cas9 handle), and a 40 nt transcription terminator derived from *S. pyogenes* (*T*_*Sp*_) [39]. *T*_*Sp*_ functioned well in *E. coli* and many other bacteria [26, 30, 39]. It was also recruited to develop CRISPRi in *C. glutamicum*, but its termination ability has not been investigated in *C. glutamicum* [3]. To evaluate *T*_*Sp*_, a *rfp* gene was inserted into the downstream of gRNA, producing plasmid pRfp3. *T*_*Sp*_ of pRfp3 was further deleted, resulting in plasmid pRfp4 (Fig. 1c). As expected, deletion of *T*_*Sp*_ increased *rfp* expression in *E. coli*, demonstrating that *T*_*Sp*_ was functional in *E. coli*. However, the *rfp* downstream *T*_*Sp*_ was highly expressed in *C. glutamicum* and deletion of *T*_*Sp*_ decreased *rfp* expression (Fig. 1d). This surprising phenomenon suggests that *T*_*Sp*_ does not function as a terminator in *C. glutamicum*. In plasmid pgRNA1, *T*_*Sp*_ was followed by a 185 bp redundant segment and a *rrnB* terminator (*T*_*rrnB*_). Since *T*_*Sp*_ could not terminate gRNA transcription in *C. glutamicum*, the 185 bp redundant segment will also be transcribed, which may affect the function of Cas9-gRNA complex (Fig. 1e). To confirm our hypothesis, the 185 bp redundant segment was removed from plasmid pgRNA1. The resultant plasmid pgRNA2 harbored a gRNA expression cassette in which *T*_*rrnB*_ closely followed *T*_*Sp*_ (Fig. 1f). Lethality test using pCas9 and pgRNA2 showed that the escape rate was reduced to 1.3 ± 0.2 × 10^−2^, making the CRISPR/Cas9-based counter-selection feasible (Fig. 1g).

### Markerless chromosomal gene deletion using CRISPR/Cas9 and plasmid-borne editing template

**Fig. 2:**
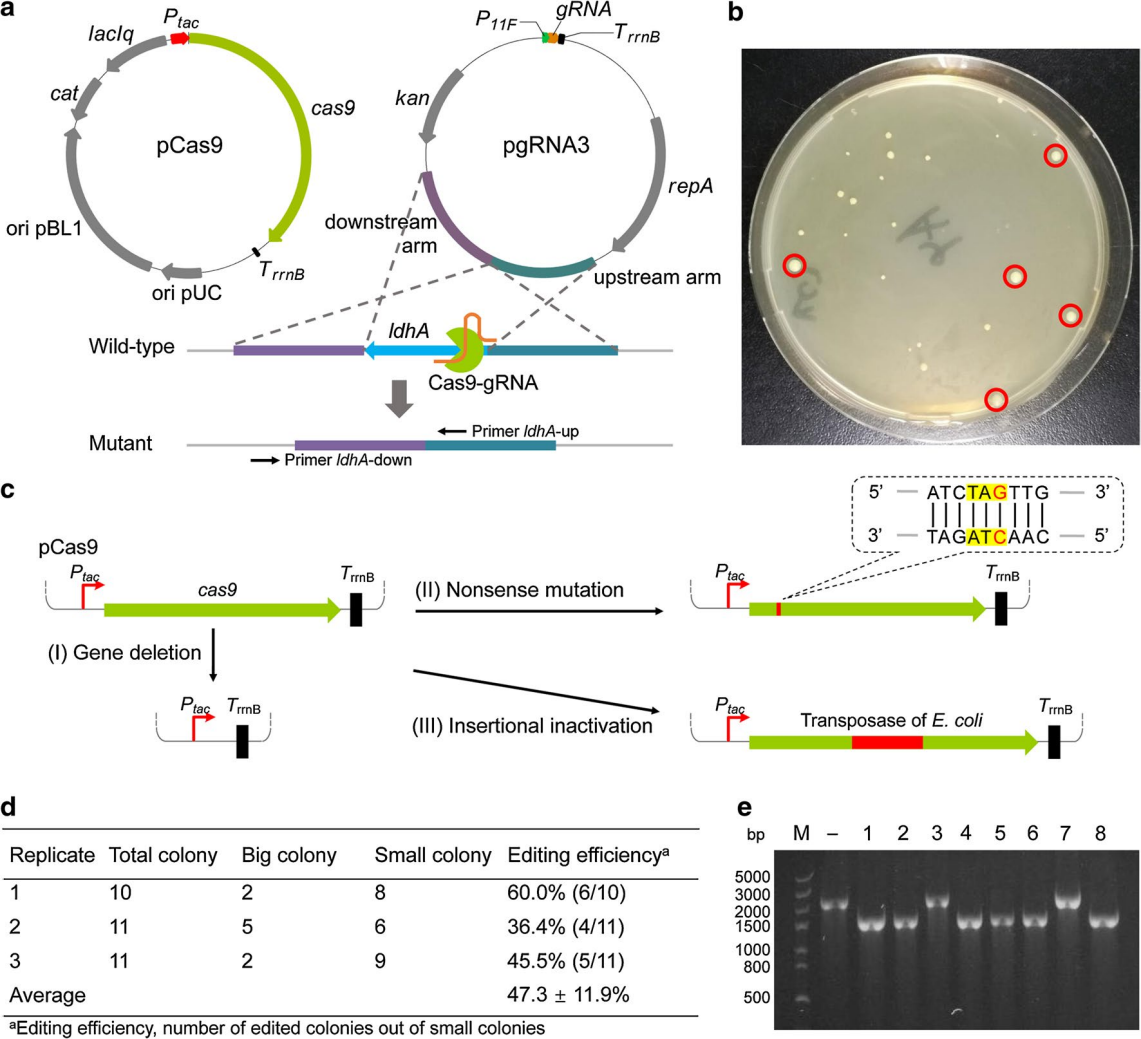
Gene deletion using CRISPR/Cas9 and plasmid-borne editing template in *C. glutamicum* SL4. **a** Schematic representation of pCas9, pgRNA3, and gene deletion event. **b** Transformants harboring pCas9 and pgRNA3 on a SGY plate supplemented with Km, Cm, and IPTG (1 mM). pCas9 and pgRNA3 were co-transformed into strain SL4 simultaneously and cells were spread on SGY plates with Km, Cm, and IPTG (1 mM) immediately after recovery. Colonies marked in red cycles were the so-called abnormally large colonies. **c** Schematic representation of different patterns of pCas9 mutation. Gene deletion, *cas9* gene was removed from pCas9. Nonsense mutation, a T465G mutation occurred, generating a stop codon (TAT to TAG) in *cas9* gene. Insertional inactivation, a transposase encoding gene from *E. coli* was inserted into *cas9* gene, which deactivated *cas9*. **d** Colony counting and editing efficiency calculation of *C. glutamicum* SL4. Colonies on SGY plates supplemented with Km, Cm, and IPTG (1 mM) were counted and verified using colony PCR. **e** PCR verification of *ldhA* deletion in *C. glutamicum* SL4 using the primer pair (*ldhA*-up and *ldhA*-down) shown in **a**. M, DNA marker; –, wild-type control; 1–8, eight small colonies. This displayed the result of Replicate 1 in **c**. The results of Replicate 2 and Replicate 3 were shown in Additional file 1: Figure S2

Since the CRISPR/Cas9 system could act as an efficient selection tool, we exploited its capacity for markerless chromosomal gene deletion. Two ~ 1000 bp homologous arms flanking at both sides of the targeted *ldhA* gene were inserted into plasmid pgRNA2. The resultant plasmid pgRNA3 was then transformed into *C. glutamicum* SL4 (pCas9) to provide a DNA editing template for deleting 664 bp of *ldhA* gene. It was expected that edited mutants would be generated through double-crossover homologous recombination and nonedited cells would be eliminated by CRISPR/Cas9-directed cleavage at the targeted *ldhA* gene (Fig. 2a). Noteworthily, the transformants obtained could be divided into two categories, abnormally large ones and small ones (Fig. 2b). Colony PCR was conducted to confirm gene deletion in the transformants using a pair of primers shown in Fig. 2a. All the abnormally large colonies were false and two of the twenty small ones tested were positive mutant (Additional file 1: Figure S1).

Although successful gene deletion was achieved by using CRISPR/Cas9 system, the editing efficiency was quite low. We speculated that two events might help the false positives escape the counter-selection. First, the targeted DNA was cleaved by Cas9 but the DSB was repaired by nucleotide or base excision repair, leading to the survival of cells and very likely some mutations at the cleavage site [40]. In this case, the targeted gene might be deactivated but PCR was not capable of verifying it. Second, pCas9 or pgRNA3 mutated, which disabled the Cas9-gRNA complex and the counter-selection system. To confirm our hypotheses, the *ldhA* genes of false positives were amplified by PCR and sequenced. pCas9 and pgRNA3 were also extracted from the false positives and sequenced. No mutation was found in gene *ldhA* or plasmid pgRNA3, whereas plasmid pCas9 mutated in several patterns as shown in Fig. 2c. In all the abnormally large colonies investigated, their *cas9* genes were all deleted from pCas9 plasmids. In the small but false colonies, their *cas9* genes were either deactivated through nonsense mutation or disrupted by insertion of a transposase encoding gene from *E. coli*. Such cells harboring mutated pCas9 escaped the counter-selection, and even worse, outgrew those expressing active Cas9, making selection of the edited cells very difficult.

Since *cas9* was easily mutated in *C. glutamicum*, reducing replication of pCas9 in the host may lessen the mutation. A one-step electrotransformation strategy was then presented that pCas9 and pgRNA3 were co-transformed into *C. glutamicum* SL4 simultaneously and cells were spread on SGY plates supplemented with Km, Cm, and IPTG (1 mM) immediately after recovery. Screening using two antibiotics and expressing *cas9* may burden cell growth, and colonies appeared after 2–3 days cultivation. We still got some abnormally large colonies and they were all proven false by colony PCR. However, the ratio of positive mutants out of all colonies obtained in one plate considerably increased, reaching an average of 47.3 ± 11.9% (Fig. 2d, e; Additional file 1: Figure S2). Given the high editing efficiency, edited cells should be easily obtained after CRISPR/Cas9 counter-selection. Meanwhile, a control experiment was conducted using pCas9 and pgRNA3-derivative plasmid that harbored no targeting spacer. As expected, no counter-selection phenomenon was observed and no gene deletion mutants were obtained (Additional file 1: Figure S3).

### Markerless chromosomal gene insertion

**Fig. 3:**
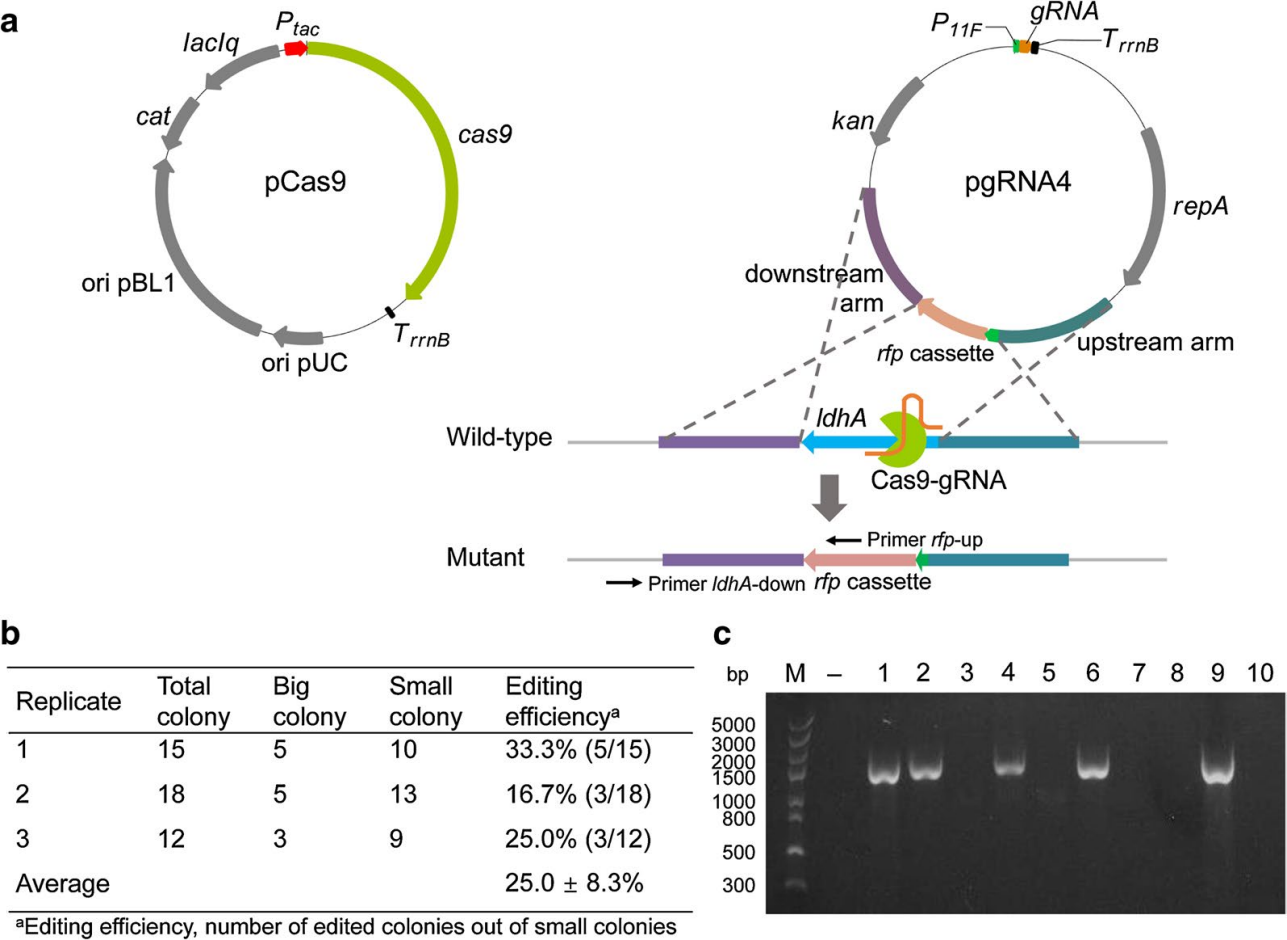
Gene insertion using CRISPR/Cas9 and plasmid-borne editing template in *C. glutamicum* SL4. **a** Schematic representation of pCas9, pgRNA4, and gene insertion event. **b** Colony counting and editing efficiency calculation of *C. glutamicum* SL4. pCas9 and pgRNA4 were co-transformed into *C. glutamicum* SL4 simultaneously and cells were spread on SGY plates supplemented with Km, Cm, and IPTG (1 mM) immediately after recovery. Colonies on the selective plates were counted and verified using colony PCR. **c** PCR verification of *rfp* insertion in *C. glutamicum* SL4 using the primer pair (*rfp*-up and *ldhA*-down) shown in **a**. M, DNA marker; –, wild-type control; 1–10, ten small colonies. This displayed the result of Replicate 1 in **b**. The results of Replicate 2 and Replicate 3 were shown in Additional file 1: Figure S4

Next, the ability of gene insertion (allelic exchange) by using CRISPR/Cas9 system was examined. A *rfp* cassette (781 bp) was inserted between the upstream and downstream homologous arms of pgRNA3. The resultant plasmid pgRNA4, together with pCas9, was co-transformed into *C. glutamicum* SL4, aiming to replace the *ldhA* gene with the *rfp* cassette (Δ664 bp and insert 781 bp) (Fig. 3a). Colony PCR was conducted to verify the gene insertion events by using a pair of primers shown in Fig. 3a. An average of 25.0 ± 8.3% of colonies were confirmed as positive mutants (Fig. 3b, c; Additional file 1: Figure S4). The mutant inserted with the *rfp* cassette in the *ldhA* gene was designated as *C. glutamicum* SL4Δ*ldhA*::*rfp*.

### ssDNA-directed recombineering using CRISPR/Cas9 and RecT

**Fig. 4:**
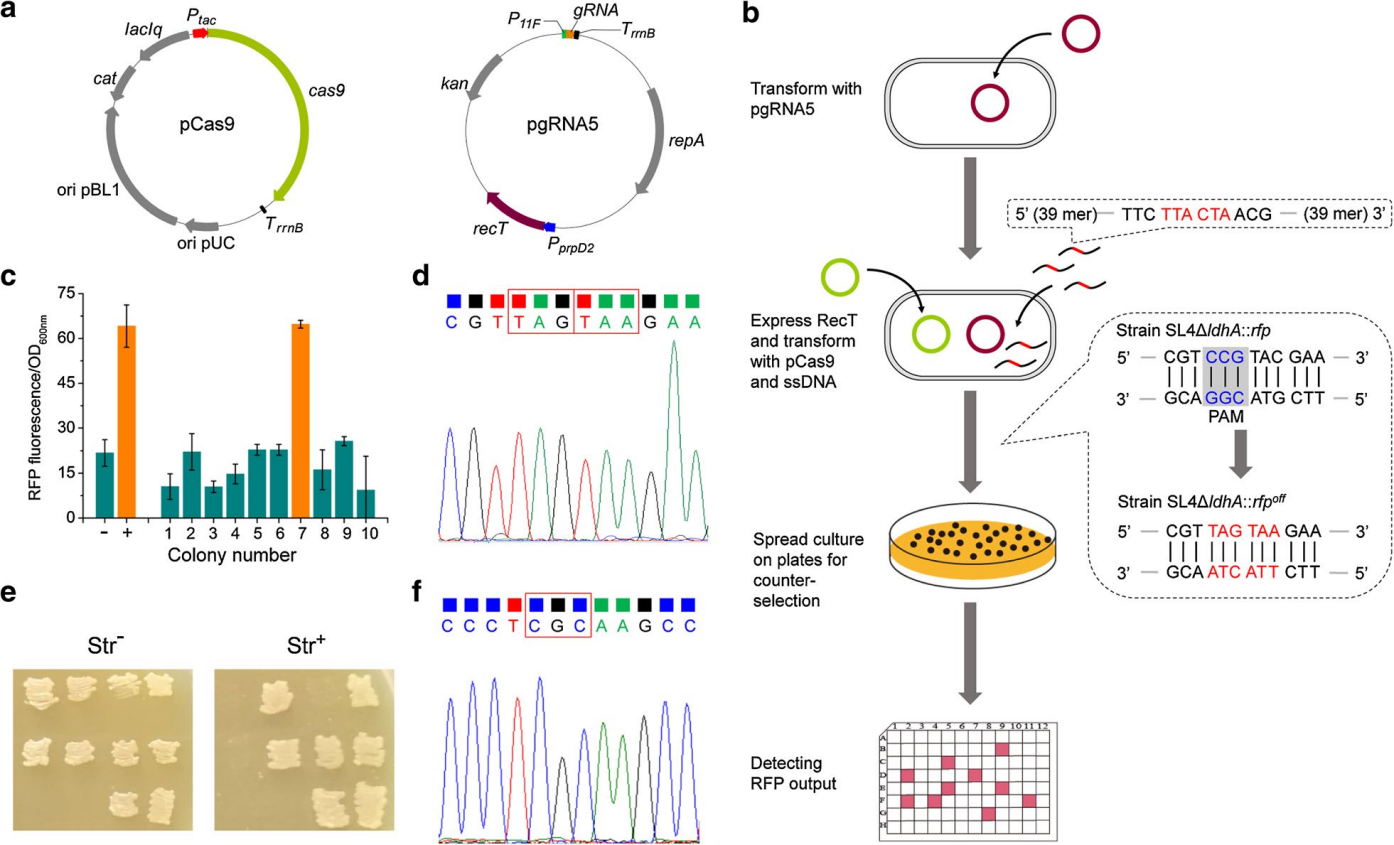
ssDNA-directed recombineering using CRISPR/Cas9 and RecT in *C. glutamicum* SL4Δ*ldhA*::*rfp*. **a** Schematic representation of pCas9 and pgRNA5 which were used for ssDNA-directed recombineering. **b** Operation scheme of ssDNA-directed recombineering using CRISPR/Cas9 and RecT for *rfp* deactivation. A 90mer ssDNA targeted to the lagging strand (*rfp*-off1, Additional file 1: Table S1) is designed to introduce two successive stop codons (highlighted in red) in *rfp* gene. PAM sequence of the gRNA is shaded grey. Plasmid pgRNA5 was first transformed into *C. glutamicum* SL4Δ*ldhA*::*rfp*. The resultant strain SL4Δ*ldhA*::*rfp* (pgRNA5) was cultivated in SGY medium supplemented with Km and sodium propionate to induce RecT expression. Electrocompetent cells were then prepared and transformed with pCas9 and ssDNA. After recovery, cells were spread on SGY plates supplemented with Km, Cm, and IPTG (1 mM) to induce Cas9 expression for counter-selection. Colonies were picked randomly and verified by measuring their fluorescence outputs. **c** Fluorescence output detection of candidate mutants of *C. glutamicum* SL4Δ*ldhA*::*rfp* ^off1^. –, wild-type *C. glutamicum* SL4 control; +, *C. glutamicum* SL4Δ*ldhA*::*rfp*; 1–10, ten colonies. This displayed the result of Replicate 1. The results of Replicate 2 and Replicate 3 were shown in Additional file 1: Figure S6. **d** *rfp* gene sequencing of *C. glutamicum* SL4Δ*ldhA*::*rfp* ^off1^ mutants. Nucleotides in red box represents the two successive stop codons (TAG TAA) introduced by ssDNA-directed recombineering. **e** Str^R^ phenotype test of *C. glutamicum* SL4 *rpsL* ^K43R^ mutants. Ten colonies were picked randomly from SGY plates supplemented with Km, Cm, and IPTG (1 mM) and patched onto SGY plates (Str^−^) and SGY plates supplemented with streptomycin (Str^+^). Seven out of ten colonies were Str^R^. **f** *rpsL* gene sequencing of *C. glutamicum* SL4 *rpsL* ^K43R^ mutants. Nucleotides in red box represents the mutated codon (AAG to CGC) introduced by ssDNA-directed recombineering

ssDNA-directed recombineering can be used to mutagenize, repair or engineer the chromosome with high efficiencies [41]. By introducing exogenous recombinases into *C. glutamicum*, the recombination efficiency using ssDNA templates was much higher than that using plasmid-borne templates [42]. However, due to lack of reliable genotype selection method, application of this technique was severely limited. Taking advantage of the CRISPR/Cas9 system developed here, counterselecting mutants generated during recombineering against wild-type cells became feasible and a ssDNA-directed gene editing tool was developed. The *recT* gene encoding recombinase RecT of prophage Rac was inserted into plasmid pgRNA4 under the control of the propionate-inducible promoter *P*_*prpD2*_, resulting in plasmid pgRNA5 (Fig. 4a). ssDNAs were designed to alter the PAM sequence or PAM-proximal 8–12 bp sequences because these regions were shown to be most crucial for Cas9 targeting specificity [43]. To obtain high recombination efficiency, 90mer ssDNAs targeted to the lagging strand were used here.

The method was first tested for the ability to introduce stop codons into a target gene. A ssDNA (*rfp*-off1, Additional file 1: Table S1) was designed to introduce two successive stop codons into the *rfp* gene of *C. glutamicum* SL4Δ*ldhA*::*rfp* (Fig. 4b). The operation scheme was shown in Fig. 4b. Plasmid pgRNA5 was first transformed into strain SL4Δ*ldhA*::*rfp*. The resultant strain SL4Δ*ldhA*::*rfp* (pgRNA5) was cultivated with addition of sodium propionate to induce RecT expression, and then electrocompetent cells were prepared. Next, cells were co-transformed with pCas9 and ssDNA, recovered, and spread on SGY plates supplemented with Km, Cm, and IPTG (1 mM) to induce Cas9 expression. Three independent experiments were conducted and hundreds of colonies could be obtained in each experiment, which were much more than the number of colonies got in gene deletion and insertion experiments (Additional file 1: Figure S5). For each replicate, 10 colonies were picked randomly and verified by measuring their fluorescence outputs, (Fig. 4c; Additional file 1: Figure S6). The gene editing events were further verified by gene sequencing (Fig. 4d), demonstrating a high editing efficiency of 86.7 ± 5.8%, an average of 90.0, 90.0, and 80.0% from three replicates. Such high mutation rate would eliminate the need of phenotype screening methods for ssDNA-directed recombineering, making genome editing of *C. glutamicum* more efficient.

Ribosomal protein S12 encoding gene *rpsL* was selected as another target for ssDNA-directed gene editing using CRISPR/Cas9. An amino acid mutation K43R (AAG to CGC in the nucleotide sequence) resulting in resistance to streptomycin (Str^R^) was described for *C. glutamicum* [44, 45]. A ssDNA was designed to mutate AAG to CGC which corresponded to amino acid mutation K43R, and plasmid pgRNA6 was then constructed. Using the protocol described previously, mutants with Str^R^ phenotype were obtained with a mutant efficiency of 70.0% (Fig. 4e). Gene sequencing further verified the targeted gene editing of *rpsL* (Fig. 4f).

### Plasmid curing

To cure the Cas9 and gRNA expression plasmids for continuous genome editing, engineered cells were cultivated in SGY medium without antibiotics for 12 h. The culture was serially transferred into fresh medium for another two times before plating on SGY plates without antibiotics. The resultant colonies were picked and tested for resistance to Km and Cm. Approximately 25.0% of colonies were sensitive to Km and Cm, confirming that both plasmids were cured. In the study that reported CRISPR/Cpf1-mediated genome editing of *C. glutamicum*, Jiang et al. replaced the replicon of pXMJ19 (*cas9* expression plasmid) with a temperature sensitive replicon pBL1^ts^ [46] and deleted the distribution protein Per encoding gene [47] in pEC-XK99E (gRNA expression plasmid), facilitating easy plasmid curing [36]. Since plasmids with the same backbones were used in our and Jiang et al.’s studies [36], the strategy reported by Jiang et al. is also applicable to optimizing the present plasmid curing procedure.

### CRISPR/Cas9-mediated genome editing in *C. glutamicum* wild-type strains

To evaluate the universality of the CRISPR/Cas9-mediated genome editing toolbox described above, its application was tested in two well-known wild-type *C. glutamicum* strains ATCC 13869 and ATCC 13032. *ldhA* gene knockout was first performed. After co-transformation of pCas9 and pgRNA3, cells were spread on SGY plates supplemented with Km, Cm, and 1 mM IPTG. However, the cell growth was severely hindered. Colonies of strain ATCC 13869 appeared after 8 days cultivation and no colonies of strain ATCC 13032 were obtained even after 10 days cultivation. Considering that expression of Cas9 can be a significant burden for cell growth, we hypothesized that the expression level of Cas9 was too high for strains ATCC 13969 and ATCC 13032. Therefore, the inducer (IPTG) usage was reduced and 0.1 mM IPTG and 0.01 mM IPTG were proven suitable for strains ATCC 13969 and ATCC 13032, respectively. After 2–3 days cultivation, colonies were picked for verification, revealing that *ldhA* deletion efficiencies reached 33.3 ± 2.5 and 60.0% in strains ATCC 13969 and ATCC 13032, respectively (Table 1). When shorter homologous arms (~ 500 bp) were used to knock out *ldhA* in strain ATCC 13032, fewer colonies but comparable deletion efficiency (50.0%) were obtained (Table 1), indicating that shorter homology arms may result in lower homologous recombination efficiencies. An 8083 bp fragment (*cgl1776*–*cgl1781*) was selected as a second target for CRISPR/Cas9-mediated gene deletion in strain ATCC 13032. Using plasmids pCas9 and pgRNA9 harboring ~ 1000 bp homologous arms, the 8083 bp fragment was successfully deleted with an efficiency of 40.0% (Table 1).

**Table 1:** CRISPR/Cas9-mediated gene deletion and insertion in *C. glutamicum* wild-type strains

Strain	Plasmids used	Deleted fragment size (bp)	Inserted fragment size (bp)	Homologous arm size (bp)	Efficiency
ATCC 13869	pCas9 and pgRNA3	664	0	~ 1000	30.8% (4/13); 35.7% (5/14); 33.3% (2/6)
	pCas9 and pgRNA4	664	781	~ 1000	28.6% (4/14)
ATCC 13032	pCas9 and pgRNA3	664	0	~ 1000	60.0% (9/15)
	pCas9 and pgRNA8	664	0	~ 500	50.0% (2/4)
	pCas9 and pgRNA9	8083	0	~ 1000	40.0% (2/5)
	pCas9 and pgRNA4	664	781	~ 1000	62.5% (5/8)
	pCas9 and pgRNA10	0	3626	~ 1000	50.0% (4/8)

By using plasmids pCas9 and pgRNA4, replacement of *ldhA* gene with the *rfp* cassette was conducted in strains ATCC 13969 and ATCC 13032. The editing efficiencies reached 28.6 and 62.5%, respectively (Table 1). Then, in order to assess the possibility of inserting larger DNA fragments into the genome of *C. glutamicum* using CRISPR/Cas9, we attempted to insert a 3626 bp *lacZ* cassette into the genomic locus between *cgl0900* and *cgl0901* without deleting any chromosomal fragment. By co-transforming pCas9 and pgRNA10 into strain ATCC 13032, insertion of the *lacZ* cassette was achieved at an efficiency of 50.0% (Table 1). These results demonstrate the possibility of deleting and inserting larger DNA fragments in *C. glutamicum*.

By using pCas9, pgRNA5 and ssDNA *rfp*-off1 (Additional file 1: Table S1), ssDNA-directed recombineering was also successfully performed in *C. glutamicum* ATCC 13032::*rfp* with an editing efficiency of 90.0% (Additional file 1: Figure S7). The achievable genome editing of different *C. glutamicum* strains with slight modification of inducer usage suggests a possible broader applicability of the CRISPR/Cas9-mediated genome editing toolbox.

### Single-nucleotide editing

Adaptive evolution and mutagenesis breeding usually generate hundreds of single nucleotide polymorphisms (SNPs) in the genome sequence. Single-nucleotide editing is a key enabling strategy to investigate the function of SNPs and assemble positive mutations (SNPs) to construct hyper producing strains. To explore the application of CRISPR/Cas9 system in single-nucleotide editing, a ssDNA (*rfp*-off2, Additional file 1: Table S1) was designed to change C to G in *rfp* gene (three bases upstream of the PAM sequence), which generated a stop codon. Similar editing efficiency (90.0%) was obtained (Additional file 1: Figure S8), demonstrating that the CRISPR/Cas9 system could be applied to precise base editing.

### Double-locus editing

**Fig. 5:**
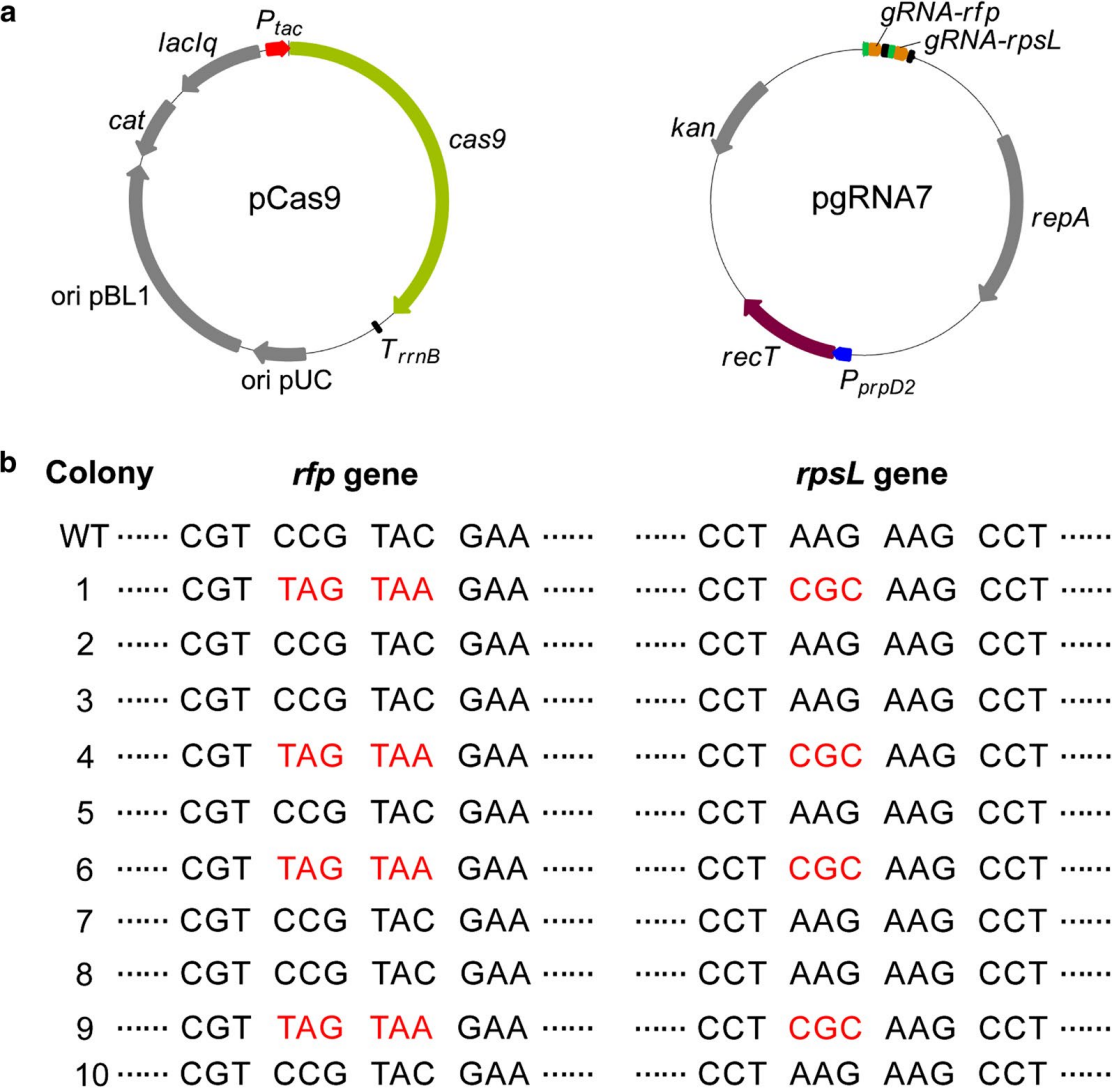
Double-locus editing in in *C. glutamicum*. **a** Schematic representation of pCas9 and pgRNA7 which were used for ssDNA-directed recombineering. **b** Mutants verification by sequencing of *rfp* and *rpsL* genes. Plasmid pgRNA7 that simultaneously expressed two gRNAs targeting *rfp* and *rpsL* was first transformed into *C. glutamicum* ATCC 13032::*rfp*. The resultant strain was then induced to express RecT and co-transformed with pCas9 and two kinds of synthetic ssDNAs (10 μg *rfp*-off1 and 10 μg *rpsL*-K43R, Additional file 1: Table S1). After recovery, cells were spread on SGY plates supplemented with Km, Cm, and IPTG (0.01 mM) to induce Cas9 expression for counter-selection. Colonies were picked and verified by gene sequencing

Since ssDNA-directed recombineering achieved considerably high editing efficiency of single-locus editing, we then doubled the number of editing targets. *rfp* and *rpsL* were selected as targets for double-locus editing and pgRNA7 that possessed two gRNA expression cassettes targeting *rfp* and *rpsL* were constructed (Fig. 5a). When *C. glutamicum* ATCC 13032::*rfp* harboring pgRNA7 was co-transformed with pCas9 and two kinds of ssDNAs (*rfp*-off1 and *rpsL*-K43R, Additional file 1: Table S1), only ten colonies were obtained, which were much fewer than those obtained in single-locus editing. All the ten colonies were verified and four of them were the expected double-mutants, demonstrating a double-locus editing efficiency of 40.0% (Fig. 5b). With the number of editing loci increased, the number of colonies obtained decreased dramatically. Therefore, simultaneous editing of more than two loci is still difficult to achieve. It is speculated that higher recombination efficiency would be helpful.

## Discussion

As a gram-positive bacterium with good genomic stability, *C. glutamicum* is more difficult to engineer than genetically tractable hosts such as *E. coli* [40, 48]. CRISPR/Cas9-mediated ssDNA recombineering was developed for deleting 400 bp chromosomal fragment in *C. glutamicum* in the time this manuscript was being prepared [35]. However, gene deletion and insertion with plasmid-borne editing templates that are key enabling techniques for reconstruction and integration of metabolic pathways are still in demand. In this study, a tailor-made CRISPR/Cas9 toolbox was developed for efficient and comprehensive engineering of *C. glutamicum*. Notably, gene deletion and insertion with plasmid-borne editing templates were efficiently implemented. Moreover, single-nucleotide editing and double-locus editing were achieved at efficiencies of 90.0 and 40.0%, respectively, which will considerably accelerate precise genome editing of *C. glutamicum*.

*S. pyogenes* Cas9 is suggested to be toxic to *C. glutamicum* and difficult to be introduced into *C. glutamicum* [35, 36]. As an alternative, *F. novicida* CRISPR/Cpf1 system was recruited to perform genetic manipulation of *C. glutamicum* recently [36]. Compared to the CRIPSR/Cpf1 tool, the present CRISPR/Cas9 tool has some distinct advantages. First, the efficiencies of CRISPR/Cas9-mediated gene deletion and insertion using plasmid-borne editing templates were higher than those obtained by CRIPSR/Cpf1. Second, the CRISPR/Cas9 system is more suitable for genome-wide scale engineering of *C. glutamicum*. Because Cpf1 utilizes a T-rich PAM and Cas9 utilizes NGG as PAM [36, 37, 49], Cas9 has considerably more editing targets in *C. glutamicum*. A bioinformatics analysis of the genome sequence of *C. glutamicum* ATCC 13032 revealed 332,289 Cas9 targets in coding sequences, which is 169.6% of the number of Cpf1 targets (195,978) (Additional file 2: Table S2). Third, Cas9 possesses better targeting specificity and can be used for precise editing, including single-nucleotide editing. Since Cpf1 cannot distinguish a single-nucleotide mismatch in seed sequence, additional synonymous mutations need to be introduced near the targeting position [36, 37]. However, even a single synonymous mutation may alter the secondary structure of the mRNA and the expression of the mutant protein [50], limiting application of the CRIPSR/Cpf1 tool in precise editing.

Considering the advantages of CRISPR/Cas9 system, attempts to introduce the *S. pyogenes cas9* gene with native codon into *C. glutamicum* were made but failed, which may be attributable to its strong expression and non-specific binding to the chromosomal DNAs [35, 36]. To reduce the translation efficiency, the *cas9* gene originally codon-optimized for atinomycetal genomes was recruited [35]. However, in this study, the *cas9* gene with native codon was successfully transformed into *C. glutamicum* and used for counter-selection. The same IPTG-inducible promoter *P*_*tac*_ but different ribosome binding sites from the previous studies were used here, which may lead to successful introduction of the *cas9* gene. The previous and present results all suggest that fine-tuning of Cas9 expression is crucial for application of CRISPR/Cas9 system in bacteria. Another unexpected crucial factor was discovered that the frequently used gRNA terminator derived from *S. pyogenes* (*T*_*Sp*_) did not terminate transcription in *C. glutamicum*. As a consequence, redundant RNA segments would be transcribed with the gRNA, which might affect the function of Cas9-gRNA complex. Deletion of the 185 bp redundant segment between *T*_*Sp*_ and plasmid-borne *T*_*rrnB*_ resulted in an acceptable escape rate (1.3 ± 0.2 × 10^−2^) for counter-selection.

During CRISPR/Cas9-mediated microbial genome editing progress, production of false positives seemed inevitable even in *E. coli*, of which the escape rate of counter-selection was quite low [11, 12, 16]. CRISPR/Cpf1-assisted editing also suffers from the same problems [36]. Although such false positives will disturb screening of edited cells, the mechanism behind the phenomenon remained unclear [16]. Here we demonstrated that modification on the *cas9* gene was the main cause of producing such false positives (Fig. 2c). In the case of CRISPR/Cpf1-assisted editing, Cpf1 mutations may also be the major reason for false positives formation since editing efficiency decreased when continuous editing was conducted using the Cpf1 expression plasmid transformed in the former round of editing [36]. By using a one-step electrotransformation strategy to reduce replication of pCas9 in the host, we alleviated the problem and increased gene deletion efficiency significantly. Previous studies suggested that small amount of Cas9 proteins produced by leaky expression can already lead to introduction of DSB resulting in SOS induction [51]. Our experiments also showed that even in the absence of gRNA, Cas9 expression hindered cell growth of *C. glutamicum*. Therefore, we hypothesized that such SOS induction in *C. glutamicum* may lead to modification of the *cas9* expression cassette. To overcome this obstacle, more strictly controlled promoter is needed for Cas9 and Cpf1 expression.

Electrotransformation and homologous recombination efficiencies of *C. glutamicum* are another two key factors that affects the acquisition of edited mutants [35]. In this study, *C. glutamicum* SL4, a derivative of strain ATCC 13869, was first selected as the target strain because of its high electrotransformation efficiency. Comparative genome analysis between strains SL4 and ATCC 13869 revealed some mutations in cell wall synthesis-related genes, such as flippase, arabinosyltransferase, and murein biosynthesis protein encoding genes. It is speculated that these mutations may contribute to the high electroporation efficiency of strain SL4 and further studies on the mechanism are underway. In some cases (e.g., gene deletion in *C. beijerinckii*), serial subculturing was applied to compensate for the low homologous recombination efficiency and enrich edited cells [26]. However, because false positives would outgrow edited cells, such strategy was unsuitable for the present case. Previous studies demonstrated that introducing exogenous recombinase or exonuclease benefited homologous recombination in *C. glutamicum* [35, 42, 52], which gave us inspiration to improve the editing efficiency of the CRISPR/Cas9 toolbox.

## Conclusions

In summary, a CRISPR/Cas9 toolbox was developed for efficient and comprehensive engineering of the industrial workhorse *C. glutamicum*. Markerless gene deletion, gene insertion, single-nucleotide editing and double-locus editing were achieved by using a customized two-plasmid-based CRISPR/Cas9 system and a simplified co-transformation strategy. This toolbox works well in several *C. glutamicum* strains and holds promise for renovating the genome editing of corynebacteria.

## Methods

### Microorganisms, cultivation conditions, and plasmids

All bacterial strains used in this study are listed in Table 2. *E. coli* DH5α was used for general cloning and cultivated aerobically at 37 °C in Luria–Bertani (LB) broth. Km (25 μg/mL) or Cm (20 μg/mL) was added to LB broth as required. *C. glutamicum* ATCC 13869 and *C. glutamicum* ATCC 13032 were obtained from the American Type Culture Collection (ATCC). *C. glutamicum* SL4 was a derivative of strain ATCC 13869 with high electrotransformation efficiency. *C. glutamicum* SL4 and its derivatives were cultivated aerobically at 30 °C in SGY medium containing 18 g/L soya peptone, 5 g/L glucose, 10 g/L yeast extract, 1 g/L K_2_HPO_4_·3H_2_O, 1 g/L urea, 0.5 g/L succinic acid, and 10 μg/L biotin. *C. glutamicum* ATCC 13032, *C. glutamicum* ATCC 13869 and their derivatives were cultivated aerobically at 30 °C in SGY medium or LBHIS medium [53]. Km (25 μg/mL), Cm (5 μg/mL), streptomycin (Str, 25 μg/mL) or IPTG (0.01 to 1 mM) was added in the medium as required. All plasmids used in this study (Table 2) were constructed using ClonExpress II One Step Cloning Kit or ClonExpress MultiS One Step Cloning Kit (Vazyme, Nanjing, China), which facilitated ligation of two or more DNA fragments through 15–20 bp overlaps. Primers and synthetic ssDNAs used for genetic manipulation are listed in Additional file 1: Table S1.

**Table 2:** Strains and plasmids used in this study

Strain or plasmid	Description^a^	Reference or source
*E. coli*		
DH5α	General cloning host	TaKaRa
MG1655	Source of *recT*	Lab collection
*C. glutamicum*		
ATCC 13869	Wild-type strain	ATCC
ATCC 13032	Wild-type strain	ATCC
ATCC 13032::*rfp*	ATCC 13032 derivative with insertion of a *rfp* cassette	Lab collection
SL4	ATCC 13869 derivative with high electrotransformation efficiency	Lab collection
SL4 (pCas9)	SL4 derivative harboring pCas9	This work
SL4Δ*ldhA*	SL4 derivative with *ldhA* deletion	This work
SL4Δ*ldhA*::*rfp*	SL4 derivative with insertion of a *rfp* cassette in *ldhA*	This work
SL4Δ*ldhA*::*rfp* (pgRNA5)	SL4Δ*ldhA*::*rfp* derivative harboring pgRNA5	This work
SL4Δ*ldhA*::*rfp* ^off1^	SL4Δ*ldhA*::*rfp* derivative with nonsense mutation in *rfp*, constructed by ssDNA (*rfp*-off1) recombineering	This work
SL4 (pgRNA6)	SL4 derivative harboring pgRNA6	This work
SL4 *rpsL* ^K43R^	SL4 derivative with K43R mutation of *rpsL*	This work
SL4 (pcas9 + pgRNA1)	SL4 derivative harboring pcas9 and pgRNA1	This work
SL4 (pcas9 + pgRNA2)	SL4 derivative harboring pcas9 and pgRNA2	This work
ATCC 13869Δ*ldhA*	ATCC 13869 derivative with *ldhA* deletion	This work
ATCC 13032Δ*ldhA*	ATCC 13032 derivative with *ldhA* deletion	This work
ATCC 13032Δ*cgl1776*-*cgl178*	ATCC 13032 derivative with *cgl1776*-*cgl178* deletion	This work
ATCC 13032::*lacZ*	ATCC 13032 derivative with *lacZ* insertion	This work
ATCC 13032::*rfp* (pgRNA5)	ATCC 13032::*rfp* derivative harboring pgRNA5	This work
ATCC 13032::*rfp* ^off1^	ATCC 13032::*rfp* derivative with nonsense mutation in *rfp*, constructed by ssDNA (*rfp*-off1) recombineering	This work
ATCC 13032::*rfp* ^off2^	ATCC 13032::*rfp* derivative with nonsense mutation in *rfp*, constructed by ssDNA (*rfp*-off2) recombineering	This work
Plasmid		
pEC-XK99E	Expression vector of *C. glutamicum*, IPTG-inducible promoter *P*_*trc*_, Km^R^	[55]
pXMJ19	Expression vector of *C. glutamicum*, IPTG-inducible promoter *P*_*tac*_, Cm^R^	[56]
pRed_Cas9_recA_Δpoxb300	Source of *cas9* gene and gRNA fragment	[54]
pEC-XK99E-*rfp*	Source of *rfp* gene	Lab collection
pCas9	pXMJ19 derivative carrying *cas9* gene, driven by IPTG-inducible promoter *P*_*tac*_	This work
pRfp1	pXMJ19 derivative carrying *cas9* ^180bp^-*rfp* artificial gene, driven by IPTG-inducible promoter *P*_*tac*_	This work
pRfp2	pXMJ19 derivative carrying *cas9* ^180bp^-*rfp* artificial gene, driven by propionate-inducible promoter *P*_*prpD2*_	This work
pRfp3	pEC-XK99E derivative carrying gRNA-*rfp* cassette, driven by constitutive promoter *P*_*11F*_	This work
pRfp4	pEC-XK99E derivative carrying gRNA (with *T*_*sp*_ deleted)-*rfp* cassette, driven by constitutive promoter *P*_*11F*_	This work
pgRNA1	pEC-XK99E derivative carrying gRNA targeting *ldhA*, driven by IPTG-inducible promoter *P*_*tac*_	This work
pgRNA2	pEC-XK99E derivative carrying gRNA targeting *ldhA*, driven by constitutive promoter *P*_*11F*_, followed by *T*_*rrnB*_ without the 185 bp redundant segment	This work
pgRNA3	pgRNA2 derivative carrying homologous arms (~ 1 kb) for *ldhA* deletion and corresponding gRNA cassette	This work
pgRNA4	pgRNA3 derivative with a *rfp* cassette inserted between the two homologous (~ 1 kb) arms for replacing *ldhA* with the *rfp* cassette	This work
pgRNA5	pgRNA2 derivative carrying gRNA targeting *rfp* and a *P*_*prpD2*_-*recT* cassette for ssDNA-directed *rfp* editing	This work
pgRNA6	pgRNA5 derivative carrying gRNA targeting *rpsL* and a *P*_*prpD2*_-*recT* cassette for ssDNA-directed *rpsL* editing	This work
pgRNA7	pgRNA5 derivative carrying two gRNAs targeting *rfp* and *rpsL* and a *P*_*prpD2*_-*recT* cassette for double-locus editing	This work
pgRNA8	pgRNA3 derivative carrying homologous arms (~ 0.5 kb) for *ldhA* deletion and corresponding gRNA cassette	This work
pgRNA9	pgRNA3 derivative carrying homologous arms (~ 1 kb) for *cgl1776*-*cgl1781* deletion (8083 bp) and corresponding gRNA cassette	This work
pgRNA10	pgRNA3 derivative carrying homologous arms (~ 1 kb), *lacZ* cassette (3626 bp) and corresponding gRNA cassette for *lacZ* cassette insertion	This work

^a^Km^R^ and Cm^R^ represent resistance to kanamycin and chloramphenicol, respectively

### Electrotransformation protocol of *C. glutamicum*

*C. glutamicum* SL4 and its derivatives cultivated overnight in SGY medium were inoculated into 100 mL fresh SGY medium to an OD_600nm_ value of 0.3. When OD_600nm_ value reached ~ 0.8, cells were collected by centrifugation at 8000 rpm and 4 °C for 10 min. After washed with ice-cold deionized distilled water for 4 times, cells were resuspended in 0.5 mL 10.0% (v/v) glycerol and 100 μL aliquots of competent cells were obtained. DNA (less than 10 μL) was added to the competence cells and transferred to a 2 mm electroporation cuvette (BioRad) with parameters set at 2500 V and 5 ms. Electroporations were performed with Eppendorf Electroporator 2510. After electroporation, 1 mL SGY medium was added immediately. Cells were incubated for 2–6 h at 30 °C, and spread on SGY plates supplemented with antibiotics and IPTG as required. The plates were incubated at 30 °C until colonies appeared. Regarding to *C. glutamicum* ATCC 13869, *C. glutamicum* ATCC 13032 and their derivatives, preparation of competent cells and electrotransformation were performed according to the protocols described previously [53].

### Construction of Cas9 and gRNA expression plasmids and determination of Cas9-induced lethality

To optimize the promoter for Cas9 expression, plasmids pRfp1 and pRfp2 were constructed. The first 180 bp of *cas9* gene and the full-length *rfp* gene were amplified by PCR from pRed_Cas9_recA_Δpoxb300 [54] and pEC-XK99E-*rfp* using primer pairs *cas9* ^180bp^-1/*cas9* ^180bp^-2 and *rfp*-1/*rfp*-2, respectively. The *cas9* ^180bp^ segment and *rfp* gene were subcloned into the *Hin*dIII and *Pst*I sites of pXMJ19 under control of *P*_*tac*_, producing pRfp1. *P*_*prpD2*_, *cas9* ^180bp^, *rfp*, and pXMJ19 backbone were amplified by PCR using primer pairs *P*_*prpD2*_-1/*P*_*prpD2*_-2, *cas9* ^180bp^-3/*cas9* ^180bp^-4, *rfp*-1/*rfp*-2, and pXMJ19-1/pXMJ19-2, respectively, and assembled into pRfp2. pRfp1 and pRfp2 were introduced into *C. glutamicum* SL4 through electrotransformation separately. The transformants were cultivated in the presence or absence of 1 mM IPTG (for pRfp1) or 1 g/L sodium propionate (for pRfp2). Cells of the stationary growth phase were used to detect their fluorescence outputs using a microplate reader (λ excitation = 560 nm, λ emission = 607 nm).

To construct plasmid pCas9, the *cas9* gene was amplified by PCR using primer pair *cas9*-1/*cas9*-2 from pRed_Cas9_recA_Δpoxb300 [54] and inserted into the *Hin*dIII and *Pst*I sites of pXMJ19 under control of *P*_*tac*_. Plasmid pgRNA1 was produced by ligating *lacI*q-*P*_*tac*_ fragment, gRNA fragment targeting *ldhA* and pEC-XK99E backbone. The three DNA fragments were amplified by PCR from pXMJ19, pRed_Cas9_recA_Δpoxb300 and pEC-XK99E using primer pairs *P*_*tac*_-1/*P*_*tac*_-2, gRNA-3/gRNA-4 and pEC-XK99E-4/pEC-XK99E-5, respectively. The gRNA targeting *ldhA* contained a base-pairing region (N24 5′-GTGGATATCCTGACCTACGCAGTG-3′), a Cas9 handle and a *S. pyogenes* terminator (*T*_*sp*_). In plasmid pgRNA1, the *rrnB* terminator (*T*_*rrnB*_) from pEC-XK99E backbone located in the185 bp downstream of *T*_*sp*_.

In order to confirm whether *T*_*sp*_ can work in *C. glutamicum*, plasmids pRfp3 and pRfp4 were constructed. pRfp3 was a derivative of pEC-XK99E carrying the gRNA (targeting *ldhA*) and *rfp* gene driven by the constitutive promoter *P*_*11F*_. *P*_*11F*_-gRNA was amplified by PCR from pgRNA1 using primer pair gRNA-1/gRNA-2. The *rfp* gene was amplified by PCR from pEC-XK99E-*rfp* using primer pair *rfp*-4/*rfp*-5, and the pEC-XK99E backbone was amplified using primer pair pEC-XK99E-1/pEC-XK99E-2. To construct pRfp4, the backbone of pRfp3 was amplified by PCR to remove the *T*_*sp*_ and *rfp* gene using primer pair pEC-XK99E-3/pEC-XK99E-1. The *rfp* gene was amplified by PCR from pRfp3 using primer pair *rfp*-5/*rfp*-6 and ligated to the backbone of pRfp3, resulting in pRfp4. pRfp3 and pRfp4 were introduced into *E. coli* MG1655 and *C. glutamicum* SL4 through electrotransformation separately. Cells of the stationary growth phase were used to detect their fluorescence outputs using a microplate reader (λ excitation = 560 nm, λ emission = 607 nm).

Plasmid pgRNA2 was derived from pgRNA1 by replacing *P*_*tac*_ with *P*_*11F*_ and deleting the 185 bp region between *T*_*sp*_ and *T*_*rrnB*_. The *P*_*11F*_-gRNA fragment was amplified by PCR from pgRNA1 using primer pair gRNA-1/gRNA-6. pgRNA1 backbone was amplified by PCR using primer pair pEC-XK99E-6/pEC-XK99E-2 to remove the 185 bp region. The two fragments were ligated to form pgRNA2.

To determine the escape rate of counter-selection, 1 μg pCas9 was transformed into *C. glutamicum* SL4. Cells were spread on SGY plates supplemented with Cm after 2 h recovery. After cultivated at 30 °C for 2 days, colonies were picked and verified by colony PCR. The correct transformant was designated as *C. glutamicum* SL4 (pCas9). Then pgRNA1 and pgRNA2 were transformed into *C. glutamicum* SL4 (pCas9) separately. Correct transformants were cultivated, diluted and spread on SGY plates containing Km and Cm, with or without IPTG (1 mM). The escape rate of counter-selection was calculated by colony counting.

### Gene deletion and insertion using CRISPR/Cas9

To provide editing template for *ldhA* deletion, two homologous arms of *ldhA* (~ 1 kb for each arm) were amplified by PCR from the genomic DNA of *C. glutamicum* SL4 using primer pairs *ldhA*-1/*ldhA*-2 and *ldhA*-3/*ldhA*-4, respectively. The backbone of pgRNA2 was amplified by PCR using primer pair pEC-XK99E-8/pEC-XK99E-9. pgRNA3 was constructed by ligating the two homologous arms and pgRNA2 backbone. Plasmid pgRNA4 was derived from pgRNA3 by inserting a *rfp* cassette between the two homologous arms of *ldhA*. The *rfp* cassette and pgRNA3 backbone were amplified by PCR using primer pairs *rfp*-7/*rfp*-8 and *ldhA*-2/*ldhA*-5, respectively. The two fragments were ligated to form pgRNA4. Plasmid pgRNA8 was derived from pgRNA3 by replacing the ~ 1 kb homologous arms with ~ 500 bp homologous arms for *ldhA* deletion. Primer pairs *ldhA*-6/*ldhA*-7 and pEC-XK99E-8/pEC-XK99E-9 were used to amplify the homologous arms and plasmid backbone from plasmid pgRNA3. The two fragments were ligated to form pgRNA8. Plasmid pgRNA9 was constructed to delete the *cgl1776*-*cgl1781* fragment (8083 bp). Primer pairs pEC-XK99E-13/pEC-XK99E-8 and pEC-XK99E-9/pEC-XK99E-14 were used to amplify two plasmid backbone fragments from plasmid pgRNA3. Primer pairs 8083-1/8083-2 and 8083-3/8083-4 were used to amplify two homologous arm fragments from the genomic DNA of *C. glutamicum* ATCC 13032. The four fragments were ligated to form pgRNA9. Plasmid pgRNA10 was constructed to insert the *lacZ* cassette (3626 bp) into the genomic locus between *cgl0900* and *cgl0901* in *C. glutamicum* ATCC 13032. Primer pairs pEC-XK99E-15/pEC-XK99E-8 and pEC-XK99E-9/pEC-XK99E-16 were used to amplify two plasmid backbone fragments from plasmid pgRNA3. Primer pairs 3626-1/3626-2 and 3626-3/3626-4 were used to amplify two homologous arm fragments from the genomic DNA of *C. glutamicum* ATCC 13032. Primer pair *lacZ*-1/*lacZ*-2 was used to amplify the *lacZ* cassette from the genomic DNA of *E. coli* MG1655. The five fragments were ligated to form pgRNA10.

Regarding to *ldhA* deletion in *C. glutamicum* SL4, 1 μg pgRNA3 was transformed into *C. glutamicum* SL4 (pCas9). Cells were recovered at 30 °C for 6 h and then spread on SGY plates supplemented with Km, Cm, and IPTG (1 mM). Plates were incubated at 30 °C for 2–3 days until colonies appeared. The *C. glutamicum* SL4Δ*ldhA* mutants were verified by colony PCR using primer pair *ldhA*-up/*ldhA*-down. PCR products of edited cells were 1604 bp, and those of wild-type cells were 2268 bp. When the one-step electrotransformation strategy was used, 3 μg pCas9 and 1 μg pgRNA3 were co-transformed into *C. glutamicum* SL4. Mutants screening and verification were performed using procedures described above. Regarding to *rfp* insertion in *C. glutamicum* SL4, the one-step electrotransformation strategy described above was used except that pCas9 and pgRNA4 were co-transformed into *C. glutamicum* SL4 and primer pair *rfp*-up/*ldhA*-down was used for PCR verification of *C. glutamicum* SL4Δ*ldhA*::*rfp* mutants. PCR products of edited cells were 1449 bp.

For gene deletion and insertion in *C. glutamicum* ATCC 13869 and ATCC 13032, pCas9 and pgRNA3 (pgRNA4, pgRNA8, pgRNA9, or pgRNA10) were co-transformed. After electroporation, 1 mL LBHIS broth was added immediately and the suspension was quickly incubated for 6 min at 46 °C [53]. Cells were then incubated for 6 h at 30 °C and subsequently spread on SGY or LBHIS plates supplemented with Km, Cm, and IPTG (0.01 or 0.1 mM), Plates were incubated at 30 °C for 2–3 days until colonies appeared. Mutants verification were performed by colony PCR using the primer pairs shown in Additional file 1: Table S1.

### CRISPR/Cas9-mediated ssDNA recombineering

Plasmid pgRNA5 was construct by inserting a *P*_*prpD2*_-*recT* cassette into pgRNA2 and replacing the base-pairing region of gRNA targeting *ldhA* with a base-pairing region targeting *rfp* (N20, 5′-GCGGTCTGGGTACCTTCGTA-3′). A part of pgRNA2 backbone was amplified by PCR using primer pair gRNA-7/pEC-XK99E-8. N20 for *rfp* was added to the backbone by primer gRNA-7. A second part of pgRNA2 backbone was amplified by PCR using primer pair pEC-XK99E-9/pEC-XK99E-10. *P*_*prpD2*_ and *recT* were amplified by PCR using primer pairs *P*_*prpD2*_-3/*P*_*prpD2*_-4 from the genomic DNA of *C. glutamicum* ATCC 13032 and *recT*-1/*recT*-2 from the genomic DNA of *E. coli* MG1655, respectively. The four fragments were ligated to form the pgRNA5. To construct plasmid pgRNA6, the backbone of pgRNA5 was amplified by PCR using primer pair pEC-XK99E-11/pEC-XK99E-12 with replacement of the base-pairing region of gRNA targeting *rfp* with a base-pairing region targeting *rpsL* (N20, 5′-AGAGCAGAGTTAGGCTTCTT-3′). Then the fragment was ligated upon itself to form the pgRNA6.

CRISPR/Cas9-mediated ssDNA recombineering was conducted using a two-step electrotransformation strategy. Firstly, 1 μg pgRNA5 was transformed into *C. glutamicum* SL4Δ*ldhA*::*rfp*. The competent cells of *C. glutamicum* SL4Δ*ldhA*::*rfp* (pgRNA5) were prepared by addition of 1 g/L sodium propionate to induce RecT recombinases expression, and then co-transformed with 1–2 μg pCas9 and 6–15 μg synthetic ssDNA (*rfp*-off1 or *rfp*-off2, Additional file 1: Table S1). Cells were recovered at 30 °C for 6 h and then spread on SGY plates supplemented with Km, Cm, and IPTG (1 mM). Plates were incubated at 30 °C for 2–3 days until colonies appeared. Recombination events were verified by detecting the fluorescence outputs of colonies. *C. glutamicum* SL4Δ*ldhA*::*rfp* ^off1^ mutants were further verified by gene sequencing. When *rpsL* was selected as a target of ssDNA recombineering, similar procedures described above were used with small modifications. Plasmid pgRNA6 was first transformed into *C. glutamicum* SL4. The competent cells of *C. glutamicum* SL4 (pgRNA6) was then transformed with pCas9 and synthetic ssDNA (*rpsL*-K43R, Additional file 1: Table S1). *C. glutamicum* SL4 *rpsL* ^K43R^ mutants were verified by Str^R^ phenotype test and gene sequencing. To perform ssDNA recombineering in *C. glutamicum* ATCC 13032, an engineered strain (ATCC 13032::*rfp*) was used and *rfp* was selected as a target. The procedures were similar with those for recombineering *C. glutamicum* SL4Δ*ldhA*::*rfp* except that 0.01 mM IPTG was used for inducing Cas9 expression.

### Double-locus editing

*rfp* and *rpsL* were selected as targets for double-locus editing in *C. glutamicum* ATCC 13032::*rfp*. Plasmid pgRNA7 that simultaneously expressed two gRNAs targeting *rfp* and *rpsL* was constructed by inserting a gRNA fragment targeting *rpsL* into pgRNA5. The gRNA fragment targeting *rpsL* was amplified by PCR using primer pair gRNA-8/gRNA-9 from pgRNA6. The pgRNA5 backbone was amplified by PCR using primer pair gRNA-10/pEC-XK99E-2. The two fragments were ligated to form pgRNA7. The procedures were similar with those for single-gene editing in *C. glutamicum* ATCC 13032 by using CRISPR/Cas9-mediated ssDNA recombineering, except that two kinds of synthetic ssDNAs (10 μg *rfp*-off1 and 10 μg *rpsL*-K43R, Additional file 1: Table S1) were transformed with pCas9 simultaneously.

### Plasmid curing

To remove Cas9 and gRNA expression plasmids from edited cells, the edited colony was inoculated into 100 mL of SGY medium without antibiotics and cultivated for 12 h. The culture was serially transferred into fresh medium with an inoculum size of 0.1% (v/v) for another two times, diluted, and spread on SGY plates without antibiotics. Colonies were confirmed as cured by determining their sensitivity to Km and Cm.

## Additional files

**Additional file 1: Figure S1.** PCR verification of *ldhA* deletion using CRISPR/Cas9 and the two-step electrotransformation strategy in *Corynebacterium glutamicum* SL4. M, DNA marker; –, wild-type control; 1–20, twenty colonies. pCas9 was firstly transformed into *C. glutamicum* SL4. pgRNA3 was then transformed into strain SL4 (pCas9) and cells were spread on SGY plates supplemented with Km, Cm and IPTG (1 mM) immediately after recovery. After cultivated at 30 °C, twenty small colonies instead of the abnormally large colonies were picked to perform colony PCR using a pair of primers *ldhA*-up and *ldhA*-down. **Figure S2.** PCR verification of *ldhA* deletion using CRISPR/Cas9 and the one-step electrotransformation strategy in *C. glutamicum* SL4. (a) Replicate 2. M, DNA marker; –, wild-type control; 1–6, six colonies. (b) Replicate 3. M, DNA marker; –, wild-type control; 1–9, nine colonies. pCas9 and pgRNA3 were co-transformed into *C. glutamicum* SL4 simultaneously and cells were spread on SGY plates supplemented with Km, Cm and IPTG (1 mM) immediately after recovery. After cultivated at 30 °C, all the small colonies on the plates were picked to perform colony PCR using a pair of primers *ldhA*-up and *ldhA*-down. **Figure S3.** PCR verification of *ldhA* deletion using pCas9 and pgRNA3-derivative plasmid that harbored no targeting spacer in *C. glutamicum* SL4. M, DNA marker; –, wild-type control; 1–20, twenty colonies. pCas9 and pgRNA3-derivative plasmid that harbored no targeting spacer were co-transformed into *C. glutamicum* SL4 simultaneously and cells were spread on SGY plates supplemented with Km, Cm and IPTG (1 mM) immediately after recovery. After cultivated at 30 °C, twenty colonies on the plates were picked to perform colony PCR using a pair of primers *ldhA*-up and *ldhA*-down. **Figure S4.** PCR verification of *rfp* insertion using CRISPR/Cas9 and plasmid-borne editing template in *C. glutamicum* SL4. (a) Replicate 2. M, DNA marker; –, wild-type control; 1–13, thirteen colonies. (b) Replicate 3. M, DNA marker; –, wild-type control; 1–9, nine colonies. pCas9 and pgRNA4 were co-transformed into *C. glutamicum* SL4 simultaneously and cells were spread on SGY plates supplemented with Km, Cm and IPTG (1 mM) immediately after recovery. After cultivated at 30 °C, all the small colonies on the plates were picked to perform colony PCR using a pair of primers *rfp*-up and *ldhA*-down. **Figure S5.** Transformants harboring pCas9, pgRNA5, and ssDNA (*rfp*-off1). Plasmid pgRNA5 was first transformed into *C. glutamicum* SL4Δ*ldhA*::*rfp*. The resultant strain SL4Δ*ldhA*::*rfp* (pgRNA5) was cultivated with addition of propionate to induce RecT expression, and then electrocompetent cells were prepared. Next, cells were transformed with pCas9 and ssDNA (*rfp*-off1) and spread on SGY plates supplemented with Km, Cm and IPTG (1 mM) immediately after recovery. **Figure S6.** Fluorescence output detection of candidate mutants of *C. glutamicum* SL4Δ*ldhA*::*rfp* ^off1^. (a) Replicate 2. –, wild-type *C. glutamicum* SL4 control; +, *C. glutamicum* SL4Δ*ldhA*::*rfp*; 1–10, ten colonies. (b) Replicate 3. –, wild-type *C. glutamicum* SL4 control; +, *C. glutamicum* SL4Δ*ldhA*::*rfp*; 1–10, ten colonies. **Figure S7.** Fluorescence output detection of candidate mutants of *C. glutamicum* ATCC 13032::*rfp* ^off1^. –, wild-type *C. glutamicum* ATCC 13032 control; +, *C. glutamicum* ATCC 13032::*rfp*; 1–10, ten colonies. **Figure S8.** Fluorescence output detection of candidate mutants of *C. glutamicum* ATCC 13032::*rfp* ^off2^. –, wild-type *C. glutamicum* ATCC 13032 control; +, *C. glutamicum* ATCC 13032::*rfp*; 1–10, ten colonies. **Table S1.** Sequences of primers and ssDNAs used in this study.

**Additional file 2: Table S2.** Analysis of PAMs for Cas9 and Cpf1 in the genome sequence of *C. glutamicum* ATCC 13032.

## Abbreviations

ssDNA: single-strand DNA
FACS: fluorescence-activated cell sorting
CRISPR: clustered regularly interspaced short palindromic repeats
Cas: CRISPR-associated proteins
dCas9: nuclease-deactivated Cas9
PAM: protospacer adjacent motif
DSB: double-strand breakage
NHEJ: endogenous nonhomologous end joining
IPTG: isopropyl-β-d-thiogalactopyranoside
CRISPRi: CRISPR interference
RFP: red fluorescent protein
SGY: soya peptone-glucose-yeast extract
Km: kanamycin
Cm: chloramphenicol
SNPs: single nucleotide polymorphisms
CDSs: coding sequences
