# Supplementary material for: Development of a CRISPR/Cas9 genome editing toolbox for Corynebacterium glutamicum
Source: Microb Cell Fact. 2017 Nov 16;16:205. doi: 10.1186/s12934-017-0815-5 (PMC5693361; doi:10.1186/s12934-017-0815-5)
Supplement: Supplementary file 1 — Additional file 1: Figure S1. PCR verification of ldhA deletion using CRISPR/Cas9 and the two-step electrotransformation strategy in Corynebacterium glutamicum SL4. M, DNA marker; –, wild-type control; 1–20, twenty colonies. pCas9 was firstly transformed into C. glutamicum SL4. pgRNA3 was then transformed into strain SL4 (pCas9) and cells were spread on SGY plates supplemented with Km, Cm and IPTG (1 mM) immediately after recovery. After cultivated at 30 °C, twenty small colonies instead of the abnormally large colonies were picked to perform colony PCR using a pair of primers ldhA-up and ldhA-down. Figure S2. PCR verification of ldhA deletion using CRISPR/Cas9 and the one-step electrotransformation strategy in C. glutamicum SL4. (a) Replicate 2. M, DNA marker; –, wild-type control; 1–6, six colonies. (b) Replicate 3. M, DNA marker; –, wild-type control; 1–9, nine colonies. pCas9 and pgRNA3 were co-transformed into C. glutamicum SL4 simultaneously and cells were spread on SGY plates supplemented with Km, Cm and IPTG (1 mM) immediately after recovery. After cultivated at 30 °C, all the small colonies on the plates were picked to perform colony PCR using a pair of primers ldhA-up and ldhA-down. Figure S3. PCR verification of ldhA deletion using pCas9 and pgRNA3-derivative plasmid that harbored no targeting spacer in C. glutamicum SL4. M, DNA marker; –, wild-type control; 1–20, twenty colonies. pCas9 and pgRNA3-derivative plasmid that harbored no targeting spacer were co-transformed into C. glutamicum SL4 simultaneously and cells were spread on SGY plates supplemented with Km, Cm and IPTG (1 mM) immediately after recovery. After cultivated at 30 °C, twenty colonies on the plates were picked to perform colony PCR using a pair of primers ldhA-up and ldhA-down. Figure S4. PCR verification of rfp insertion using CRISPR/Cas9 and plasmid-borne editing template in C. glutamicum SL4. (a) Replicate 2. M, DNA marker; –, wild-type control; 1–13, thirteen colonies. (b) Replicate 3 [file 12934_2017_815_MOESM1_ESM.docx]

**Additional file 1**

**Development of a CRISPR/Cas9 genome editing toolbox for *Corynebacterium glutamicum***

Jiao Liu^1,2†^, Yu Wang^1,2†^, Yujiao Lu^1,2^, Ping Zheng^1,2*^, Jibin Sun^1,2*^ , Yanhe Ma^2^

^1^Key Laboratory of Systems Microbial Biotechnology, Chinese Academy of Sciences, Tianjin 300308, People’s Republic of China

^2^Tianjin Institute of Industrial Biotechnology, Chinese Academy of Sciences, Tianjin 300308, People’s Republic of China

^*^Correspondence: zheng_p@tib.cas.cn; sun_jb@tib.cas.cn

^†^Jiao Liu and Yu Wang contributed equally to this work.

**
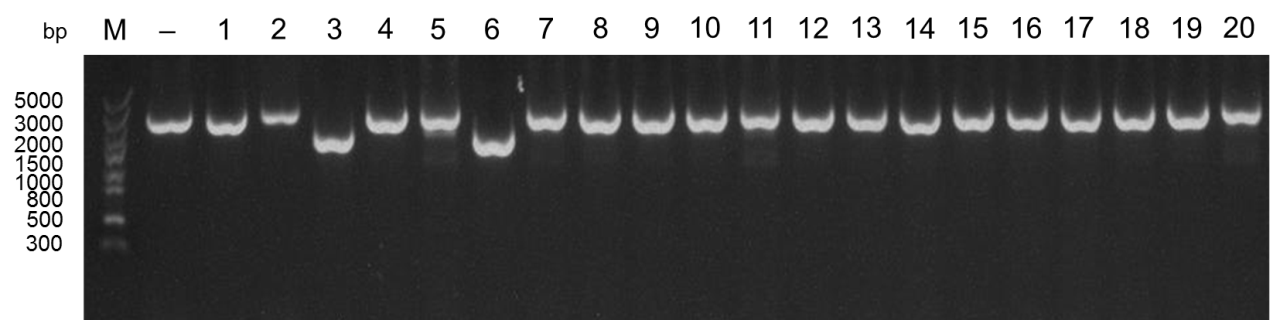
**

**Fig. S1.** PCR verification of *ldhA* deletion using CRISPR/Cas9 and the two-step electrotransformation strategy in *Corynebacterium glutamicum* SL4. M, DNA marker; –, wild-type control; 1–20, twenty colonies. pCas9 was firstly transformed into *C. glutamicum* SL4. pgRNA3 was then transformed into strain SL4 (pCas9) and cells were spread on SGY plates supplemented with Km, Cm and IPTG (1 mM) immediately after recovery. After cultivated at 30°C, twenty small colonies instead of the abnormally large colonies were picked to perform colony PCR using a pair of primers *ldhA*-up and *ldhA*-down.


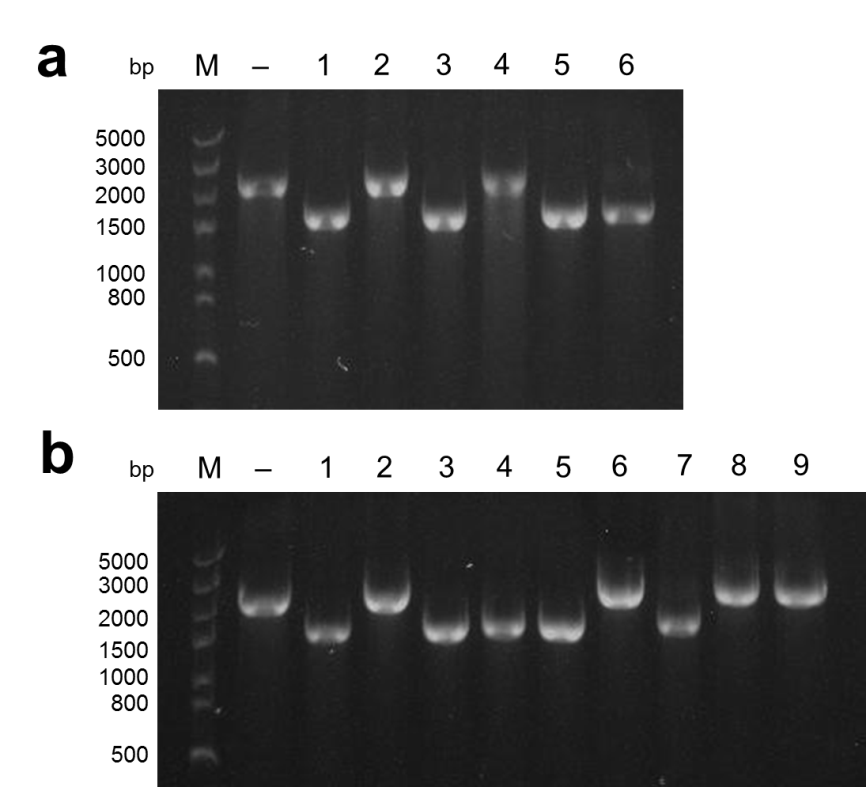


**Fig. S2.** PCR verification of *ldhA* deletion using CRISPR/Cas9 and the one-step electrotransformation strategy in *C. glutamicum* SL4. (a) Replicate 2. M, DNA marker; –, wild-type control; 1–6, six small colonies. (b) Replicate 3. M, DNA marker; –, wild-type control; 1–9, nine small colonies. pCas9 and pgRNA3 were co-transformed into *C. glutamicum* SL4 simultaneously and cells were spread on SGY plates supplemented with Km, Cm and IPTG (1 mM) immediately after recovery. After cultivated at 30°C, all the small colonies on the plates were picked to perform colony PCR using a pair of primers *ldhA*-up and *ldhA*-down.


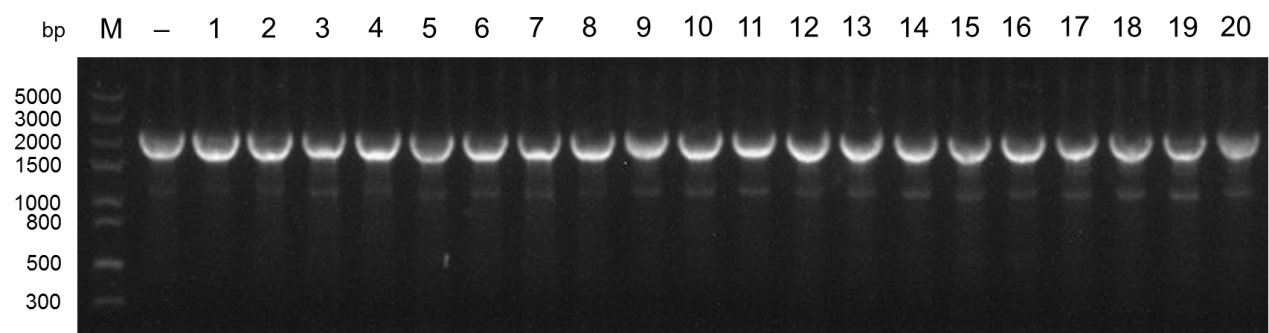


**Fig. S3.** PCR verification of *ldhA* deletion using pCas9 and pgRNA3-derivative plasmid that harbored no targeting spacer in *C. glutamicum* SL4. M, DNA marker; –, wild-type control; 1–20, twenty colonies. pCas9 and pgRNA3-derivative plasmid that harbored no targeting spacer were co-transformed into *C. glutamicum* SL4 simultaneously and cells were spread on SGY plates supplemented with Km, Cm and IPTG (1 mM) immediately after recovery. After cultivated at 30°C, twenty colonies on the plates were picked to perform colony PCR using a pair of primers *ldhA*-up and *ldhA*-down.


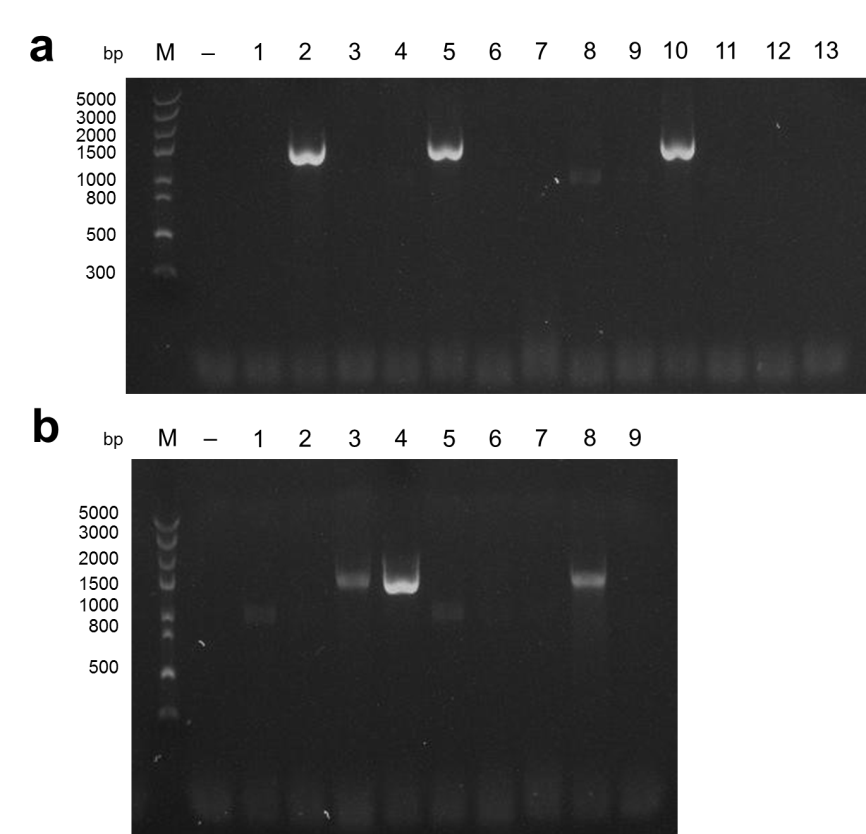


**Fig. S4.** PCR verification of *rfp* insertion using CRISPR/Cas9 and plasmid-borne editing template in *C. glutamicum* SL4. (a) Replicate 2. M, DNA marker; –, wild-type control; 1–13, thirteen small colonies. (b) Replicate 3. M, DNA marker; –, wild-type control; 1–9, nine small colonies. pCas9 and pgRNA4 were co-transformed into *C. glutamicum* SL4 simultaneously and cells were spread on SGY plates supplemented with Km, Cm and IPTG (1 mM) immediately after recovery. After cultivated at 30°C, all the small colonies on the plates were picked to perform colony PCR using a pair of primers *rfp*-up and *ldhA*-down.
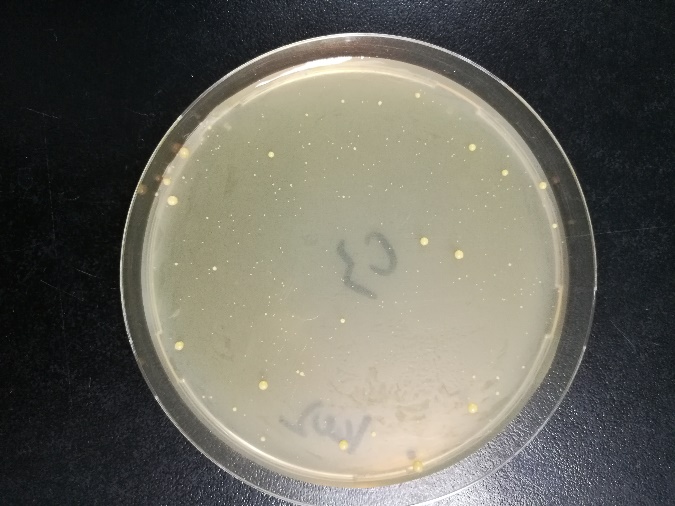


**Fig. S5.** Transformants harboring pCas9, pgRNA5, and ssDNA (*rfp*-off1). Plasmid pgRNA5 was first transformed into *C. glutamicum* SL4Δ*ldhA*::*rfp*. The resultant strain SL4Δ*ldhA*::*rfp* (pgRNA5) was cultivated with addition of propionate to induce RecT expression, and then electrocompetent cells were prepared. Next, cells were transformed with pCas9 and ssDNA (*rfp*-off1) and spread on SGY plates supplemented with Km, Cm and IPTG (1 mM) immediately after recovery.


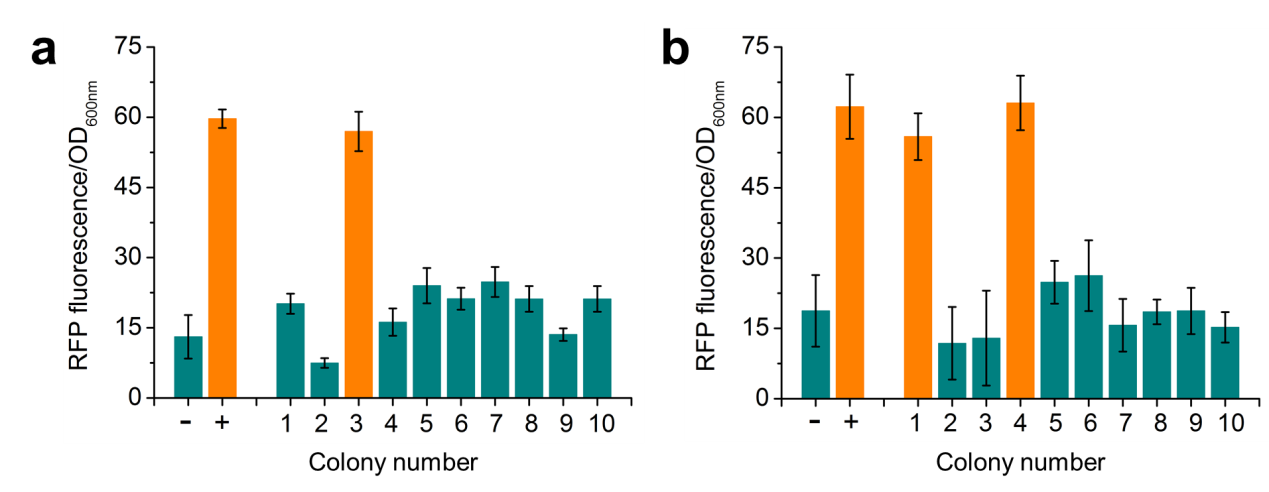


**Fig. S6.** Fluorescence output detection of candidate mutants of *C. glutamicum* SL4Δ*ldhA*::*rfp*^off1^. (a) Replicate 2. –, wild-type *C. glutamicum* SL4 control; +, *C. glutamicum* SL4Δ*ldhA*::*rfp*; 1–10, ten colonies. (b) Replicate 3. –, wild-type *C. glutamicum* SL4 control; +, *C. glutamicum* SL4Δ*ldhA*::*rfp*; 1–10, ten colonies.


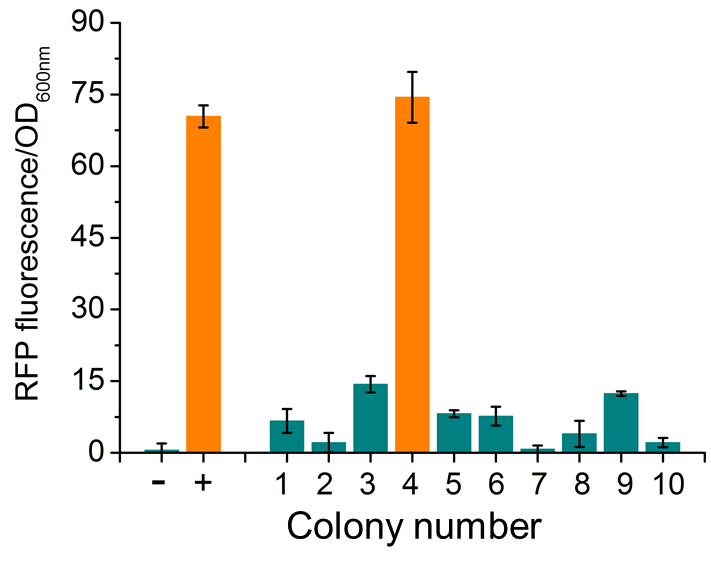


**Fig. S7.** Fluorescence output detection of candidate mutants of *C. glutamicum* ATCC 13032::*rfp*^off1^. –, wild-type *C. glutamicum* ATCC 13032 control; +, *C. glutamicum* ATCC 13032::*rfp*; 1–10, ten colonies.





**Fig. S8.** Fluorescence output detection of candidate mutants of *C. glutamicum* ATCC 13032::*rfp*^off2^. –, wild-type *C. glutamicum* ATCC 13032 control; +, *C. glutamicum* ATCC 13032::*rfp*; 1–10, ten colonies.

**Table S1. Sequences of primers and ssDNAs used in this study**

| **Oligonucleotide** | **Sequences (5′-3′)** | **Relevance** |
| --- | --- | --- |
| *cas9*-1 | GAAACAGAATTAATTAAGCTTAAAGGAGTTGAGAATGGATAAGAAATACTCAATAGGC | pCas9 construction |
| *cas9*-2 | CCTCTAGAGTCGACCTGCAGCGACTCGGTGCCACTTTTTC |  |
| *cas9*^180bp^-1 | GAAACAGAATTAATTAAGCTTAAAGGAGTTGAGAATGGATAAGAAATACTCAATAGGC | pRfp1 construction |
| *cas9*^180bp^-2 | TTCCGCTGTCTCTCCACTGTC |  |
| *rfp*-1 | ACAGTGGAGAGACAGCGGAAATGGCTTCCTCCGAAGACGTTATC |  |
| *rfp*-2 | CCTCTAGAGTCGACCTGCAGTTAAGCACCGGTGGAGTGACGAC |  |
| pXMJ19-1 | AATTCAGCTTGGCTGTTTTGGCGG | pRfp2 construction |
| pXMJ19-2 | CGCCGTTAACCACCATCAAACAGG |  |
| *P_prpD2_*-1 | TTTGATGGTGGTTAACGGCGCTCCAGCGTCCAAGAATATGCC |  |
| *P_prpD2_*-2 | TATCCATTCTCAACTCCTTTGCACACCACACTAATTCTTTAAAAAGC |  |
| *cas9*^180bp^-3 | AAAGGAGTTGAGAATGGATAAGAAATACTCAATAGGC |  |
| *cas9*^180bp^-4 | TTCCGCTGTCTCTCCACTGTC |  |
| *rfp*-1 | ACAGTGGAGAGACAGCGGAAATGGCTTCCTCCGAAGACGTTATC |  |
| *rfp*-3 | CAAAACAGCCAAGCTGAATTTTAAGCACCGGTGGAGTGACGAC |  |
| pEC-XK99E-1 | CGCAGAAGCGGTCTGATAAAAC | pRfp3 construction |
| pEC-XK99E-2 | GCCAGCTTATGTGGAGAAAACGGTGTGAAATACCGCACAGATG |  |
| *rfp*-4 | AGATTTTCAGCCTGATACAGAAAGGAGTTGAGAATGGCTTCCTCCGAAGACGTTATC |  |
| *rfp*-5 | TTTATCAGACCGCTTCTGCGTTAAGCACCGGTGGAGTGACGAC |  |
| gRNA-1 | TTTTCTCCACATAAGCTGGCAATGTTGCGACGCAACAGGTACAGTGTAATTCAGTGGATATCCTGACCTACGCAGTG |  |
| gRNA-2 | CTGTATCAGGCTGAAAATCTTCTCTC |  |
| pEC-XK99E-3 | CTAGCTCTAAAACAGGATTTCCGG | pRfp4 construction |
| pEC-XK99E-1 | CGCAGAAGCGGTCTGATAAAAC |  |
| *rfp*-6 | AAATCCTGTTTTAGAGCTAGAAAGGAGTTGAGAATGGCTTCCTCCGAAGACGTTATC |  |
| *rfp*-5 | TTTATCAGACCGCTTCTGCGTTAAGCACCGGTGGAGTGACGAC |  |
| *P_tac_*-1 | GCCAGGGTGGTTTTTCTTTTC | pgRNA1  construction |
| *P_tac_*-2 | CACTGCGTAGGTCAGGATATCCACGTGTGAAATTGTTATCCGCTC |  |
| gRNA-3 | ATATCCTGACCTACGCAGTGGTTTTAGAGCTAGAAATAGCAAG |  |
| gRNA-4 | AAAAAAAGCACCGACTCGGTGCCAC |  |
| pEC-XK99E-4 | ACCGAGTCGGTGCTTTTTTTGGCGGATGAGAGAAGATTTTCAG |  |
| pEC-XK99E-5 | AAAAGAAAAACCACCCTGGCCGGTGTGAAATACCGCACAGATG |  |
| gRNA-1 | TTTTCTCCACATAAGCTGGCAATGTTGCGACGCAACAGGTACAGTGTAATTCAGTGGATATCCTGACCTACGCAGTG | pgRNA2 construction |
| gRNA-6 | TGATGCCTGGAAAAAAAGCACCGACTCGGTGC |  |
| pEC-XK99E-6 | TGCTTTTTTTCCAGGCATCAAATAAAACGAAAGGC |  |
| pEC-XK99E-2 | GCCAGCTTATGTGGAGAAAACGGTGTGAAATACCGCACAGATG |  |
| *ldhA*-1 | AAGGGCTGCTAAAGGAAGCGTTTCATACGACCACGGGCTACC | pgRNA3 construction |
| *ldhA*-2 | GCGTATGCAACTCCGACATCG |  |
| *ldhA*-3 | GATGTCGGAGTTGCATACGCACCCGCGCAATCCTACAAAAC |  |
| *ldhA*-4 | TCTGCGGACTGGCTTTCTACTGACTTCAATCGGCAGAGCG |  |
| pEC-XK99E-8 | CGCTTCCTTTAGCAGCCCTTG |  |
| pEC-XK99E-9 | GTAGAAAGCCAGTCCGCAGAAACG |  |
| *ldhA*-2 | GCGTATGCAACTCCGACATCG | pgRNA4  construction |
| *ldhA*-5 | ACCCGCGCAATCCTACAAAAC |  |
| *rfp*-7 | GATGTCGGAGTTGCATACGCTTTTCTCCACATAAGCTGGCAATG |  |
| *rfp*-8 | TTTTGTAGGATTGCGCGGGTTTAAGCACCGGTGGAGTGACGAC |  |
| gRNA-7 | GCGGTCTGGGTACCTTCGTAGTTTTAGAGCTAGAAATAGCAAG | pgRNA5  construction |
| pEC-XK99E-8 | CGCTTCCTTTAGCAGCCCTTG |  |
| *P_prpD2_*-3 | AAGGGCTGCTAAAGGAAGCGCTCCAGCGTCCAAGAATATGCC |  |
| *P_prpD2_*-4 | ATTTGCATATCTATATCTCCTTGCACACCACACTAATTCTTTAAAAAGC |  |
| *recT*-1 | AAGGAGATATAGATATGCAAATGACTAAGCAACCACCAATCGCAAAAG |  |
| *recT*-2 | TCTGCGGACTGGCTTTCTACTACACCGCCAGGCTGAATTATTC |  |
| pEC-XK99E-9 | GTAGAAAGCCAGTCCGCAGAAACG |  |
| pEC-XK99E-10 | TACGAAGGTACCCAGACCGCTGAATTACACTGTACCTGTTGCGTC |  |
| pEC-XK99E-11 | AGAGCAGAGTTAGGCTTCTTGTTTTAGAGCTAGAAATAGCAAG | pgRNA6  construction |
| pEC-XK99E-12 | AAGAAGCCTAACTCTGCTCTTGAATTACACTGTACCTGTTGCGTC |  |
| gRNA-8 | TTTTCTCCACATAAGCTGGCAATG | pgRNA7  construction |
| gRNA-9 | TCGAAGCCGCACGTCATCTAG |  |
| gRNA-10 | TAGATGACGTGCGGCTTCGATTTTCTCCACATAAGCTGGCAATG |  |
| pEC-XK99E-2 | GCCAGCTTATGTGGAGAAAACGGTGTGAAATACCGCACAGATG |  |
| *ldhA*-6 | AAGGGCTGCTAAAGGAAGCGAACAATGACGGCGAGAGAGC | pgRNA8  construction |
| *ldhA*-7 | TCTGCGGACTGGCTTTCTACAATGCATGAGGTTGCGGG |  |
| pEC-XK99E-8 | CGCTTCCTTTAGCAGCCCTTG |  |
| pEC-XK99E-9 | GTAGAAAGCCAGTCCGCAGAAACG |  |
| 8083-1 | AAGGGCTGCTAAAGGAAGCGTACTCCCGGAAATAGCTCTCCATC | pgRNA9  construction |
| 8083-2 | TACTCCCGGAAATAGCTCTC |  |
| 8083-3 | ATTGTGTCCACATTTTCTGGTTGCCTACCGAAACTACTGACTCAC |  |
| 8083-4 | TCTGCGGACTGGCTTTCTACCATTTCCTGGTAGTAGCCTTCGG |  |
| pEC-XK99E-13 | ACTCACTGCTAACCAGCACAGTTTTAGAGCTAGAAATAGCAAG |  |
| pEC-XK99E-8 | CGCTTCCTTTAGCAGCCCTTG |  |
| pEC-XK99E-9 | GTAGAAAGCCAGTCCGCAGAAACG |  |
| pEC-XK99E-14 | TGTGCTGGTTAGCAGTGAGTTGAATTACACTGTACCTGTTGCGTC |  |
| 3626-1 | AAGGGCTGCTAAAGGAAGCGATATGCTCGATAGCCACGGGAAAC | pgRNA10  construction |
| 3626-2 | CTGAGAGAGTTGCAGCAAGCGGGCAGGCCATAGAATCGCG |  |
| 3626-3 | GCTGTTTGGTCTGCTTTCTGAGCCTCTGACCGATGTCTCGACTC |  |
| 3626-4 | TCTGCGGACTGGCTTTCTACTGGTCAATCACCTGGGCATCTC |  |
| *lacZ*-1 | GCTTGCTGCAACTCTCTCAGG |  |
| *lacZ*-2 | CAGAAAGCAGACCAAACAGCG |  |
| pEC-XK99E-15 | ACATCGGTCAGAGGCTGGGCGTTTTAGAGCTAGAAATAGCAAG |  |
| pEC-XK99E-8 | CGCTTCCTTTAGCAGCCCTTG |  |
| pEC-XK99E-9 | GTAGAAAGCCAGTCCGCAGAAACG |  |
| pEC-XK99E-16 | GCCCAGCCTCTGACCGATGTTGAATTACACTGTACCTGTTGCGTC |  |
| *rfp*-off1 | ACCACCTTTGGTAACTTTCAGTTTAGCGGTCTGGGTACCTTCTTACTAACGACCTTCACCTTCACCTTCGATTTCGAACTCGTGACCGTT | *rfp* nonsense mutation |
| *rfp*-off2 | ACCACCTTTGGTAACTTTCAGTTTAGCGGTCTGGGTACCTTCCTACGGACGACCTTCACCTTCACCTTCGATTTCGAACTCGTGACCGTT |  |
| *rpsL*-K43R | AGGTAAGGCGCACACGAGCGACCTTACGAAGAGCAGAGTTAGGCTTGCGAGGGGTGGTGGTGTACACACGGGTGCATACGCCACGACGCT | *rpsL* K43R mutation |
| *ldhA*-up | TCTGCAGGGCATAGATTGGTTTTG | Δ*ldhA* verification |
| *ldhA*-down | CCGATTTTGAGGTTGATGCG |  |
| *rfp*-up | TACAAAACCGACATCAAACTGGAC | Δ*ldhA::rfp* verification |
| *ldhA*-down | CCGATTTTGAGGTTGATGCG |  |
| 8083-F | AGCAAAGGCAGCATTGACAGAG | Δ*cgl1776*-*cgl1781* verification |
| 8083-R | TCGCCAGATCGTTTCATTGC |  |
| 3626-F | AGGAGTGCGTGTCAGACGAATG | *lacZ* cassette insertion verification |
| 3626-R | AGCCAGATTCGCAAGGGTC |  |
| *rfp*-A | CAAAGTTCGTATGGAAGGTTCC | *rfp* sequencing |
| *rfp*-B | CTTCCGGGTACATACGTTCG |  |
| *rpsL*-A | GCCAACTATTCAGCAGCTGGTC | *rpsL*  sequencing |
| *rpsL*-B | TTGCGGTCCTTAACACCCTG |  |
